# Deep phenotyping the right ventricle to establish translational MRI biomarkers for characterization of adaptive and maladaptive states in pulmonary hypertension

**DOI:** 10.1038/s41598-024-79029-3

**Published:** 2024-11-30

**Authors:** Nicoleta Baxan, Lin Zhao, Ali Ashek, Marili Niglas, Dingyi Wang, Fatemeh Khassafi, Farah Sabrin, Olivier Dubois, Chien-Nien Chen, Soni Savai Pullamsetti, Martin Wilkins, Lan Zhao

**Affiliations:** 1 National Heart and Lung Institute, Faculty of Medicine, Imperial College London, Hammersmith Hospital, London, UK; 2 Biological Imaging Centre, Imperial College London, Hammersmith Hospital, London, UK; 3 Department of Lung Development and Remodeling, Max Planck Institute for Heart and Lung Research, Member of the German Center for Lung Research (DZL), Member of the Cardio-Pulmonary Institute (CPI), Bad Nauheim, Germany; 4 Department of Internal Medicine, Universities of Giessen and Marburg Lung Center, Member of the DZL and CPI, Justus Liebig University, Giessen, Germany

**Keywords:** Adaptive/maladaptive right ventricle, MRI, Myocardial extracellular matrix, Pulmonary artery blood flow, Pulmonary hypertension, RV phenotyping, Biomarkers, Cardiology

## Abstract

Deep phenotyping the right ventricle (RV) is essential for understanding the mechanisms of adaptive and maladaptive RV responses to pulmonary hypertension (PH). In this study, feature selection coupled with machine learning classification/ranking of specific cardiac magnetic resonance imaging (MRI) features from cine-MRI, flow-sensitized, and extracellular-volume techniques were used to assess RV remodelling in monocrotaline (MCT) and Sugen hypoxia (SuHx) PH rats. Early physiological changes associated with RV adaptation were detected along with prediction of RV maladaptive outcomes. Key adaptation features included haemodynamic alterations of pulmonary blood flow ejection and wave reflection, mild RV dilatation, progressive RV hypertrophy with subtle extracellular volume growth of RV wall. A dominant component of maladaptation was the extracellular matrix increase at RV insertion points and septum, observations compatible with histopathologic and RNA-sequencing results. The upregulation of mammalian target of rapamycin (mTOR) paralleled by AMP-activated protein kinase (AMPK) deactivation was seen at 4-week MCT and 8-week SuHx, along with reduced sarcoplasmic/endoplasmic reticulum Ca^2+^ATPase (SERCA2) expression, strongly associated with the RV systolic malfunction seen at this stage in vivo. The here established MRI features can serve as potential imaging biomarkers to evaluate PH treatment efficacy in preclinical studies and build up translational markers for the PH clinic.

## Introduction

The function of right ventricle (RV) is recognized as a significant independent determinant of survival of patients with various forms of pulmonary hypertension (PH)^1–3^. As progressive RV dysfunction is associated with poor patient outcome^4^, improving the understanding of RV’s adaptive capability and recognizing early mechanisms that potentiate adverse remodelling are important in the management of the disease. Deep phenotyping of the RV through the assessment of its complex functional and structural response to PH, is essential for characterizing disease progression as well as evaluating the development of new treatments. There is a critical necessity to establish non-invasive biomarkers and molecular pathways that mediate the deterioration from adaptive to maladaptive RV remodelling and predict RV failure^5^.

The complex shape of the RV poses challenges for accurate phenotyping. The RV wall is thinner and more compliant than the left ventricle (LV)^6^ and its stroke work is a quarter that of the LV despite similar stroke volumes^7^. To date, the advances made in cardiac magnetic resonance imaging (MRI) enabled acquisition of high-resolution images of the heart^8^ for serial non-invasive and well-defined morphological and functional assessments of LV and RV. Cardiac MRI is a safe and reliable modality that has been used in the PH clinic to capture cardiac remodelling processes such as: ventricular hypertrophy^9^, RV dilatation^10^, systolic dysfunction^11^, velocity alterations in the pulmonary vascular system^12^, and myocardial fibrosis^13^, aiming to understand remodelling mechanisms intrinsic to the RV.

Cardiac MRI had also been applied in experimental animal models, offering accurate non-invasive heart assessment to explore the underlying pathophysiological mechanisms of disease with a higher level of rigor as compared to human. In this study, we sought to establish a standardized framework that incorporates a combination of carefully selected cardiac MRI parameters to characterize and distinguish the adaptive RV state as well as to predict maladaptation in two of the most used experimental PH rat models: monocrotaline (MCT)^14^ and Sugen 5416 in combination with chronic hypoxia (SuHx)^15^. Both models mimic relevant (if not all) hemodynamic, histopathological and pathophysiological processes reflective of human PH phenotypes^16^.

Through the application of regularized feature selection alongside supervised machine learning classification and ranking, we have identified the optimal subset of MRI parameters capturing longitudinally throughout the PH development in rats, as most relevant to indicate early RV adaptive physiological changes, as well as to characterize and predict maladaptive outcomes. These findings were validated through comparison with histological and molecular assays. The selected features designating RV dilatation and hypertrophy, in conjunction with either pulmonary arterial blood flow or myocardial extracellular volume alterations, may serve as potential noninvasive translational biomarkers for understanding and validating the cardiac remodelling processes in PH patients. The predictive model built using these key parameters creates a unique MRI platform for phenotyping RV behavior in rodent PH models. This is particularly valuable for assessing RV-targeted therapeutic strategies and elucidating the underlying mechanisms that sustain RV function or prevent/reverse RV failure.

## Results

### Establishment of the pulmonary hypertension phenotype in MCT and SuHx animal models

#### Haemodynamic assessment

Compared to healthy controls, MCT (4-weeks) and SuHx (8-weeks) rats presented significantly increased mean pulmonary arterial pressure (mPAP, MCT 38.0 ± 9.41 mmHg, SuHx 51.5 ± 6.78 mmHg vs. 17.5 ± 3.66 mmHg in controls) and increased RV systolic pressures at endpoints (Fig. S2 Supplement). Systolic blood pressure (SBP) remained unchanged.

#### Functional and structural changes of the right and left ventricle

**Fig. 1:**
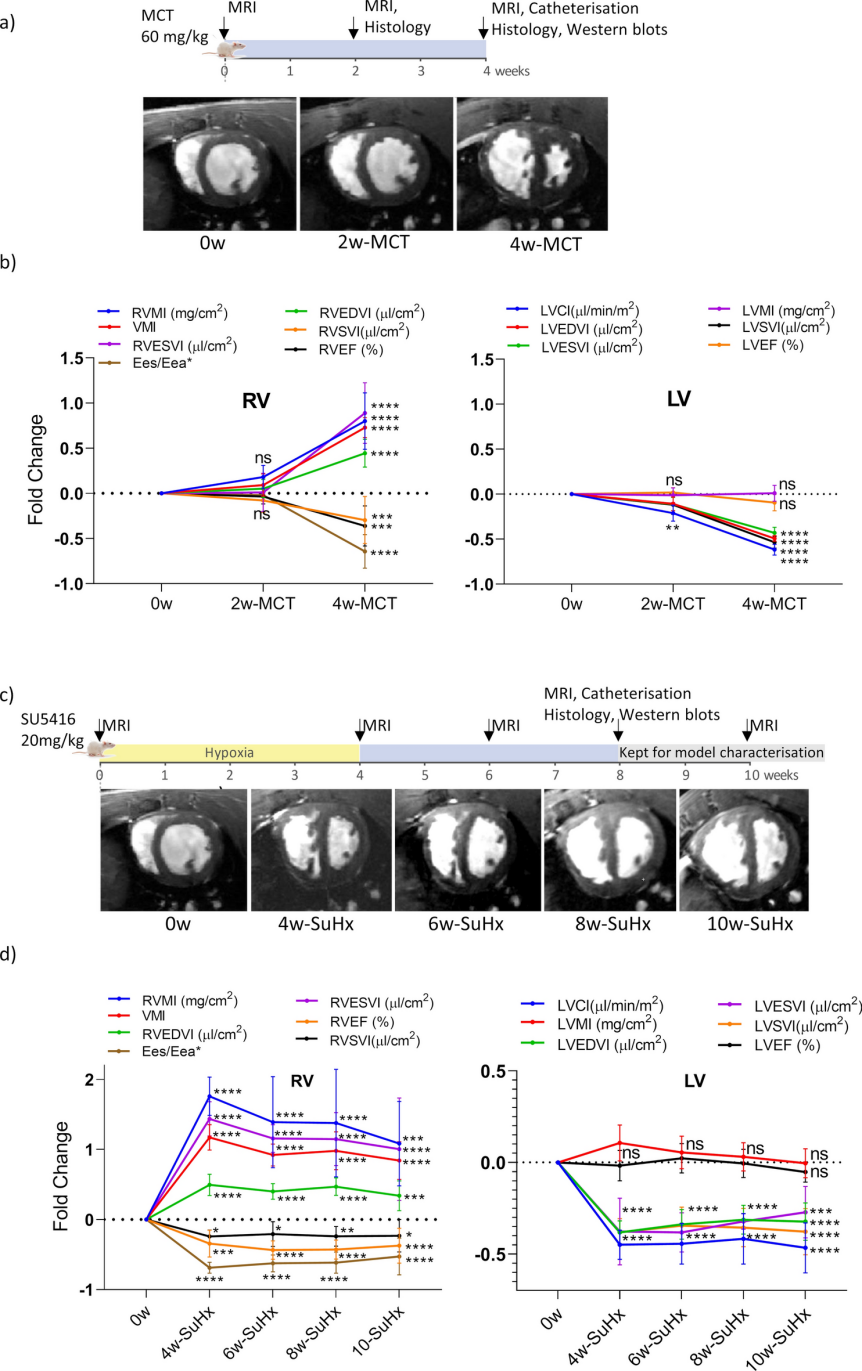
Longitudinal assessment of RV and LV in male rats. (**a**) Experimental design and representative cardiac MRI images acquired at 0, 2-, and 4-weeks MCT (**a**) and 4-, 6-, 8-, 10-week SuHx (**c**). Cardiac MRI derived metrics to assess hypertrophy, dilatation, and systolic function of RV and LV in MCT (**b**) and SuHx rats (**d**). Results are presented as fold change from baseline values (mean ± SD, *p < 0.05, **p < 0.01, ***p < 0.001, ****p < 0.0001, one-way ANOVA, comparison to healthy controls, Dunnett correction for multiple comparisons): 0-weeks (n = 12), 2-weeks MCT (n = 12), 4-weeks MCT (n = 9), 4-weeks SuHx (n = 7), 6-weeks SuHx (n = 15), 8-weeks SuHx (n = 10), 10-weeks SuHx (n = 10).

Cardiac MRI demonstrated significant structural and functional changes in MCT and SuHx rats compared with healthy controls. Representative short-axis mid-ventricular images from healthy control and pathological MCT and SuHx timepoints are illustrated in Fig. 1a,c. Specifically, in the 2-week MCT rat, there were very mild increases in RVMI, VMI, and RVEDVI with maintained RVESVI, RVSV, RVEF and RVESV/RVSV (surrogate coupling index Eea/Ees^*^^17^). Milder decreases in LVCI, LVEDVI, LVESVI were observed alongside maintained LVEF and LVSVI. 4-week MCT showed prominent RV dilatation, RVH and septal flattening that induced a D-shape of the ventricles. The RV stroke volume index, ejection fraction and Eea/Ees^*^ were significantly reduced. While LVCI deteriorated significantly at 4-weeks, this effect did not impact the LV ejection fraction. No LV hypertrophy was observed in the MCT cohorts. The MCT results are summarised in Fig. 1b.

SuHx rats developed prominent RVH, and slightly milder RV dilatation compared to 4-week MCT. RV dysfunction, present throughout all scanned timepoints, was indicated by reduced RVEF, RVSVI and Eea/Ees^*^. LV compression and prominent LVCI reduction was observed, interestingly, without impacting the LVEF. No LV hypertrophy was noted in the SuHx cohorts. At all cardiac indices, except RV hypertrophy, 10-week SuHx values displayed higher variability. The SuHx results are summarised in Fig. 1d.

### PAAT shortening and mid-systolic notching are markers of early pathological changes in PA blood flow

**Fig. 2:**
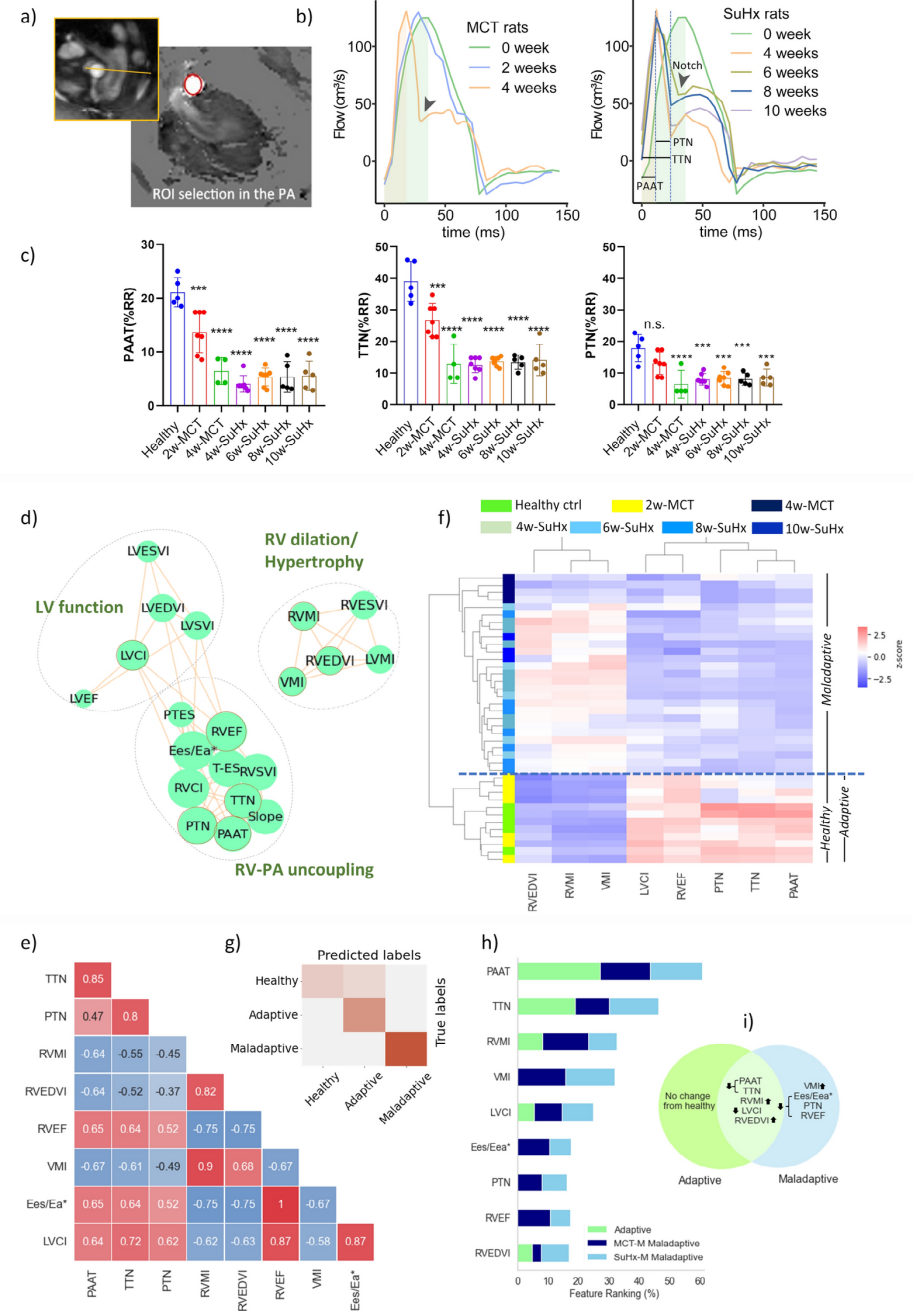
PA haemodynamic alterations in MCT and SuHx rats and correlation with RV function. (**a**) MRI planning including ROI selection outlining the PA on the phase contrast image, scale bar 2.5 mm (**b**) PA blood velocity curves of MCT and SuHx rats at specified time points. Arrow shows systolic notching. (**c**) PAAT, TTN and PTN assessment for MCT and SuHx rats. (mean ± SD, *p < 0.05, **p < 0.01, ***p < 0.001, ****p < 0.0001, baseline (n = 5), 2-weeks MCT (n = 7), 4-weeks MCT (n = 4), 4-weeks SuHx (n = 7), 6-weeks SuHx (n = 7), 8-weeks SuHx (n = 5), 10-weeks SuHx (n = 5), one-way ANOVA, compared to healthy, Dunnett correction for multiple comparisons). (**d**) correlation network revealing three distinct clusters. (**e**) correlation matrix of selected features. (**f**) dendrogram and heat map of hierarchical clustering analysis. Of note, one 10-week SuHx rat was found as part of the adaptive cluster (data not shown). (**g**) Confusion matrix build from healthy/adaptive and maladaptive classes. (**h**) ranking of selected features contribution to classify the adaptive/maladaptive states. (**i**) Venn diagram highlighting common and distinct features of adaptive/maladaptive stages.

Representative images of the MRI planning set to cross-section the pulmonary artery, the ROI selection outlining the PA and the corresponding phase contrast image are presented in Fig. 2a. Typical PA blood velocity curves are shown in Fig. 2b. PAAT was found as one of the first parameters to change in the 2-week MCT rat. PAAT shortening was further associated with the appearance of a systolic notch (arrow Fig. 2b). ‘Notching’ was marginally noticeable at 2-week MCT, prominent at 4-week MCT and all SuHx timepoints and occurred earlier in systole compared to healthy controls. This observation was quantitatively confirmed by the significantly reduced time to notch (TTN) and peak to notch (PTN) parameters to half fold of baseline values (Fig. 2c).

### PAAT and TTN shortening correlate with RV dysfunction

A correlation-based network was built from all cine- and flow MRI derived parameters (Fig. 2d). Network analysis of positive correlations identified distinct clusters related to (1) ventricular mass and RV dilatation, (2) RV function/PA blood flow characteristics and (3) LV function. Strong correlations between PAAT, TTN, PTN with RV hypertrophy, RV dilatation, RVEF and Eea/Ees* were observed (correlation matrix, Fig. 2e).

Using the mRMR FS algorithm, MRI metrics were selected based on their relevance to the target variable. LASSO regularisation further identified the subset of MR features that is most optimal for MCT and SuHx timepoint classification. A combination of eight metrics (features)—majority RV driven—were obtained. Interestingly, the selected features were linked with alterations in PA blood flow (PAAT, TTN, PTN), RV hypertrophy (RVMI, VMI), RV dilatation (RVEDVI), RV function (RVEF) and LV function (LVCI). The dendrogram and heat map from the hierarchical clustering conducted on the selected features revealed two clusters: cluster 1 (lower half of the dendrogram) consisted of healthy controls and 2-week MCT rats, and cluster 2 (upper half of the dendrogram) included the 4-week MCT and SuHx rats (Fig. 2f).

The 2-weeks MCT rats of cluster 1 displayed morphometric and physiological traits similar to those of the healthy group, particularly showing maintained systolic RV function (i.e. preserved RVEF, Eea/Ees* and LVCI). However, features such as: shortened PAAT and TTN, mild hypertrophy, and minimal RV dilatation suggested subtle remodelling, indicative of RV adaptation^18^. Along with declines in PAAT, TTN and PTN, cluster 2 exhibited pronounced hypertrophy, RV dilatation, a decline in systolic function and ventriculo-arterial uncoupling, indicating established signs of RV maladaptation^19^. These observations lead us to designate the 2-weeks MCT rats of cluster 1 as adaptive, whilst the rats belonging to cluster 2 were designated maladaptive.

The random forest classifier built on the selected features (1) ranked the importance (%) of selected features contribution to the adaptive and maladaptive classes and (2) evaluated the classification performance of the FS method. The classifier attributed the highest rank to PAAT, TTN shortening and RVMI increase (contributions of 20.2%, 15.3%, and 10.9%, respectively). Indeed, these parameters had changed considerably in the adaptive stage and remained pathologically altered during the maladaptive state. They were followed by VMI increase, reduced LVCI, declined RVEF and Eea/Ees*, shortened PTN and increased RVEDVI. RVMI was moderately higher in maladaptive MCT compared to SuHx, whereas RVEDVI had a marginally higher rank in maladaptive SuHx compared to MCT. The accuracy of the here proposed classification was assessed using a confusion matrix build for healthy, adaptive, and maladaptive profiles (Fig. 2g). In general, the data demonstrated distinct separation between healthy/adaptive and maladaptive classes of MCT and SuHx rats. Overall, the maladaptive class was 100% correctly predicted (accuracy 100%, AUC = 100%), whilst few misclassified cases were found between the healthy and adaptive groups (accuracy 92%, AUC = 91%). The summary of feature ranking (%) and corresponding Venn diagram is presented in Fig. 2h,i.

### ECV enhancement is a relevant feature at the RV insertion points, septum and RV free wall

**Fig. 3:**
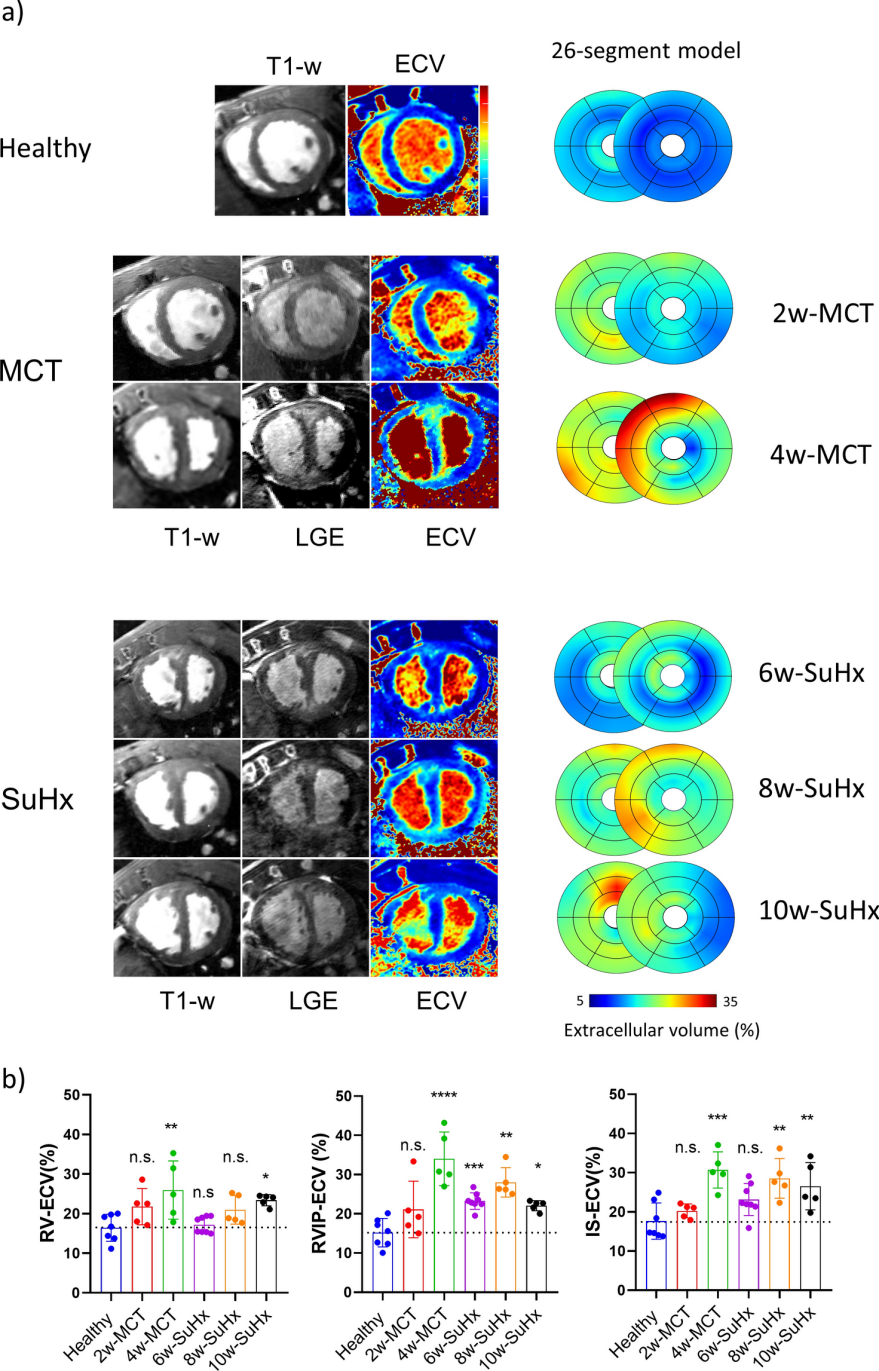
Extracellular volume imaging of RV and LV. (**a**) Representative T_1_ weighted, LGE and ECV images of healthy control, 2-, 4-week MCT and 6-, 8-, 10-SuHx (left) alongside average ECV values mapped regionally on the 26-segment bullseye plots (right). (**b**) ECV values at RV wall (RV-ECV), RV insertion points (RVIP-ECV), and septum (IS-ECV) at all imaged timepoints (mean ± SD, *p < 0.05, **p < 0.01, ***p < 0.001, ****p < 0.0001, baseline (n = 7), 2-weeks MCT (n = 5), 4-weeks MCT (n = 5), 6-weeks SuHx (n = 8), 8-weeks SuHx (n = 5), 10-weeks SuHx (n = 5), one-way ANOVA—comparison to healthy, Dunnett correction for multiple comparisons).

Representative T_1_ weighted, LGE and ECV images of healthy control, 2-week, 4-week MCT and SuHx time points are illustrated in Fig. 3a (left) alongside the average ECV values mapped regionally on the 26-segment model bullseye plots (right).

ECV mapping provided insights into cardiac tissue characteristics and indicated increased extracellular volume in the RV wall, in addition to the septum and ventricular insertion regions. Specifically, at 2-weeks MCT, ECV showed a mild increase in the RV wall and at the anterior basal RVIP. Significant increases in IS, RVIP and LV wall were found at 4-week MCT. SuHx rats exibited similar ECV behaviour, albeit with a lower intensity level than the 4-week MCT (Fig. 3b).

### Increased extracellular volume correlates with RVH and RV dysfunction

**Fig. 4:**
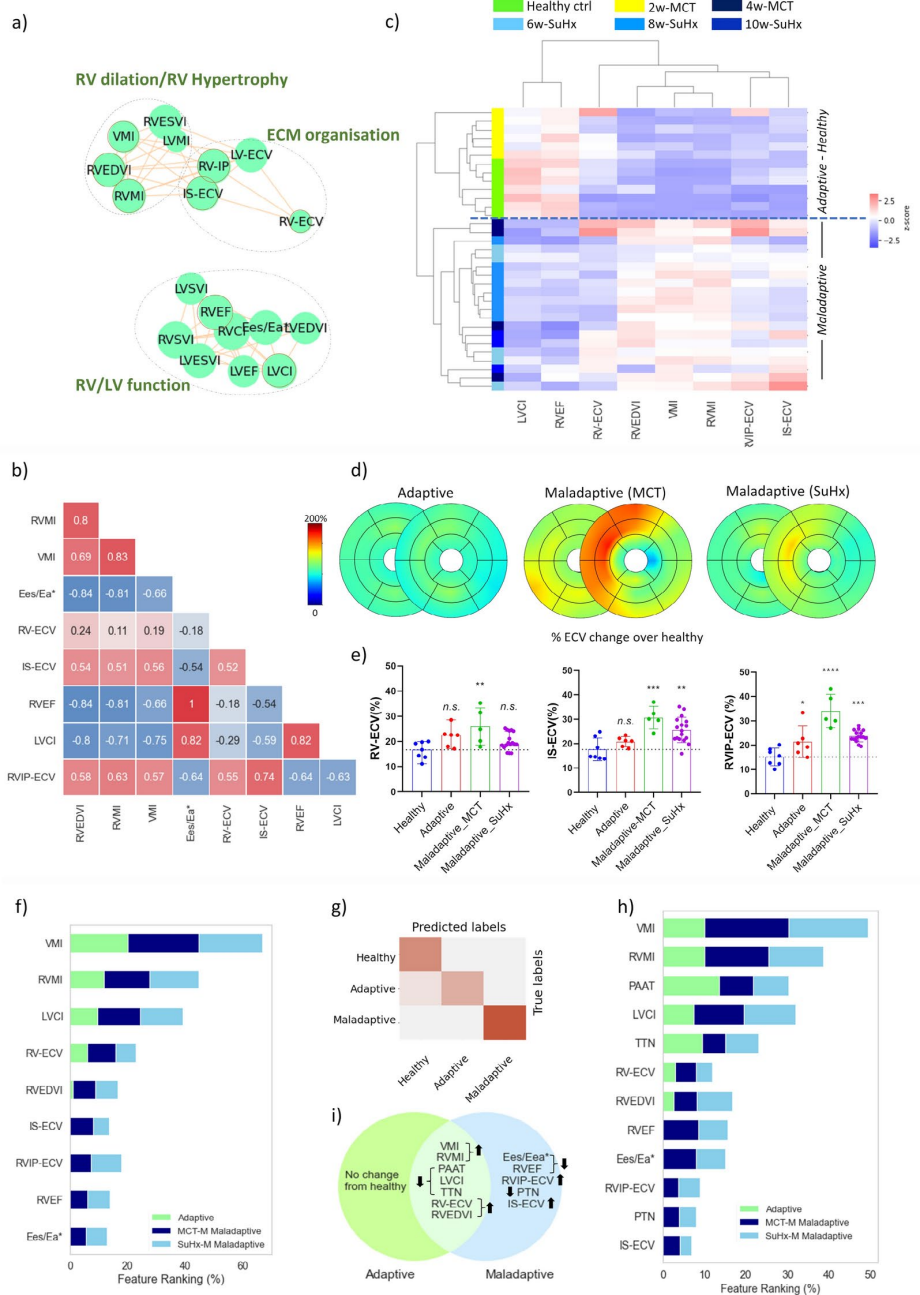
Relationship of extracellular volume with RV function and structure (**a**) Correlation network revealing three distinct clusters. (**b**) Correlation matrix of selected features. (**c**) Dendrogram and heat map of hierarchical clustering analysis. Of note, one 10-week SuHx rat was found in the adaptive cluster (not shown). (**d**) ECV bullseye plots showing ECV changes at adaptive/maladaptive stage. (**e**) ECV at RV wall (RV-ECV), RV insertion points (RVIP-ECV), and septum (IS-ECV) at healthy, adaptive, and maladaptive MCT and SuHx rats (mean ± SD, *p < 0.05, **p < 0.01, *** p < 0.001, **** p < 0.0001, healthy (n = 7), adaptive (n = 6), maladaptive MCT (n = 5), maladaptive SuHx (n = 17), one-way ANOVA—comparison to healthy, Dunnett correction for multiple comparisons). (**f**) ranking of selected features contribution to classify the adaptive and maladaptive MCT and SuHx rats. (**g**) Healthy/adaptive and maladaptive classes. Due to high similarity, all 10-week SuHx cases were classified into either 6-week or 8-week SuHx timepoints. (**h**) Ranking of selected features summarising both experiments (**i**) Venn diagram highlighting common and distinct features of adaptive/maladaptive stage.

The correlation-based network (Fig. 4a) revealed distinct interactions clustered into signatures of (1) RV hypertrophy/RV dilation (2) ECM organisation, and (3) RV-LV functional changes.

Increased ECV (particularly at RVIP and IS) correlated well with measures of RVH, dilatation, and RV dysfunction (Fig. 4b). High correlation of IS-ECV with RVEDVI (0.58, p < 0.01) and RVIP-ECV with RVEF (0.65, p < 0.01) were observed.

The selected features were related in this case with ECM organisation of RV wall and septum (RV-ECV, RVIP-ECV, IS-ECV), RV hypertrophy (RVMI, VMI), RV dilatation (RVEDVI), RV function (RVEF) and LV function (LVCI). The dendrogram and heat map from the hierarchical clustering analysis highlighted two distinct clusters: cluster 1 made of healthy controls and 2-week MCT rats, and cluster 2 which included 4-week MCT and SuHx rats (Fig. 4c). Despite being similar to the healthy group, the 2-week MCT rats in Cluster 1 exhibited mild abnormalities associated with increased extracellular volume in the RV wall, moderate dilatation and slight decline in LVCI, all of which suggested a cardiac adaptability phenotype^20^. Cluster 2 displayed notable RV hypertrophy and dilatation, accompanied by decreased systolic function and ventriculoarterial uncoupling, pronounced fibrosis at interventricular septum and insertion points, traits indicative of RV maladaptation.

Regional ECV changes at adaptive and maladaptive states are summarised in Fig. 4d,e. The random forest classifier attributed the highest rank to VMI and RVMI increase (contribution of 22.25 and 14.9%, respectively), followed by reduced LVCI, increased RV-ECV, RVIP-ECV, IS-ECV, RV-EDVI, lower RVEF and lower Eea/Ees* (Fig. 4f). IS-ECV was higher in maladaptive MCT compared to SuHx, whilst RVIP-ECV had marginally higher rank in maladaptive SuHx compared to MCT. The confusion matrix (Fig. 4g) proved distinct separation between the healthy/adaptive and maladaptive MCT and SuHx classes. The maladaptive class was 100% correctly predicted (accuracy and AUC = 100%), whilst few misclassified cases were found in the healthy and adaptive groups (accuracy 92%, AUC = 90%). Ranking of selected features from both experiments is presented in Fig. 4h and corresponding Venn diagram in Fig. 4i.

### Sample size considerations

The minimum sample size required to detect significant changes indicative of RV adaptation compared to healthy, as well as changes associated with maladaptation compared to adaptive and healthy states, revealed that several selected MRI features are strong candidates to effectively discriminate the different RV stages with high statistical power. Firstly, the severe RV phenotype observed at maladaptive stage, allowed all selected MRI features to distinguish RV maladaptation from healthy controls with a relatively small sample size (n = 3–6) and a statistical power of 95%.

#### Adaptive from healthy

Flow derived parameters such as PAAT, TTN could detect RV adaptive state from healthy with a minimum sample size of n = 4 and 95% statistical power, while RVMI and LVCI required a minimum n = 6 to reach the same statistical power. RV-ECV required a sample size of n = 6 to yield 90% statistical power, whereas PTN required n = 5 for 85% power.

#### Maladaptive from adaptive

PAAT, TTN could distinguish the maladaptive from adaptive state with a minimum sample size of n = 4 and a 95% statistical power, whereas for IS-ECV, a sample size of n = 5 with 90% statistical power was needed. For PTN, minimum sample size of n = 4 with 85% power was required. Morphometric and functional parameters such as VMI, RVMI, RVEDVI, LVCI, Ees/Eea* necessitated a minimum sample size of 3–5 to achieve 95% statistical power.

### Fibrosis colocalizes with increased ECV, hypertrophy and reduced capillary density

**Fig. 5:**
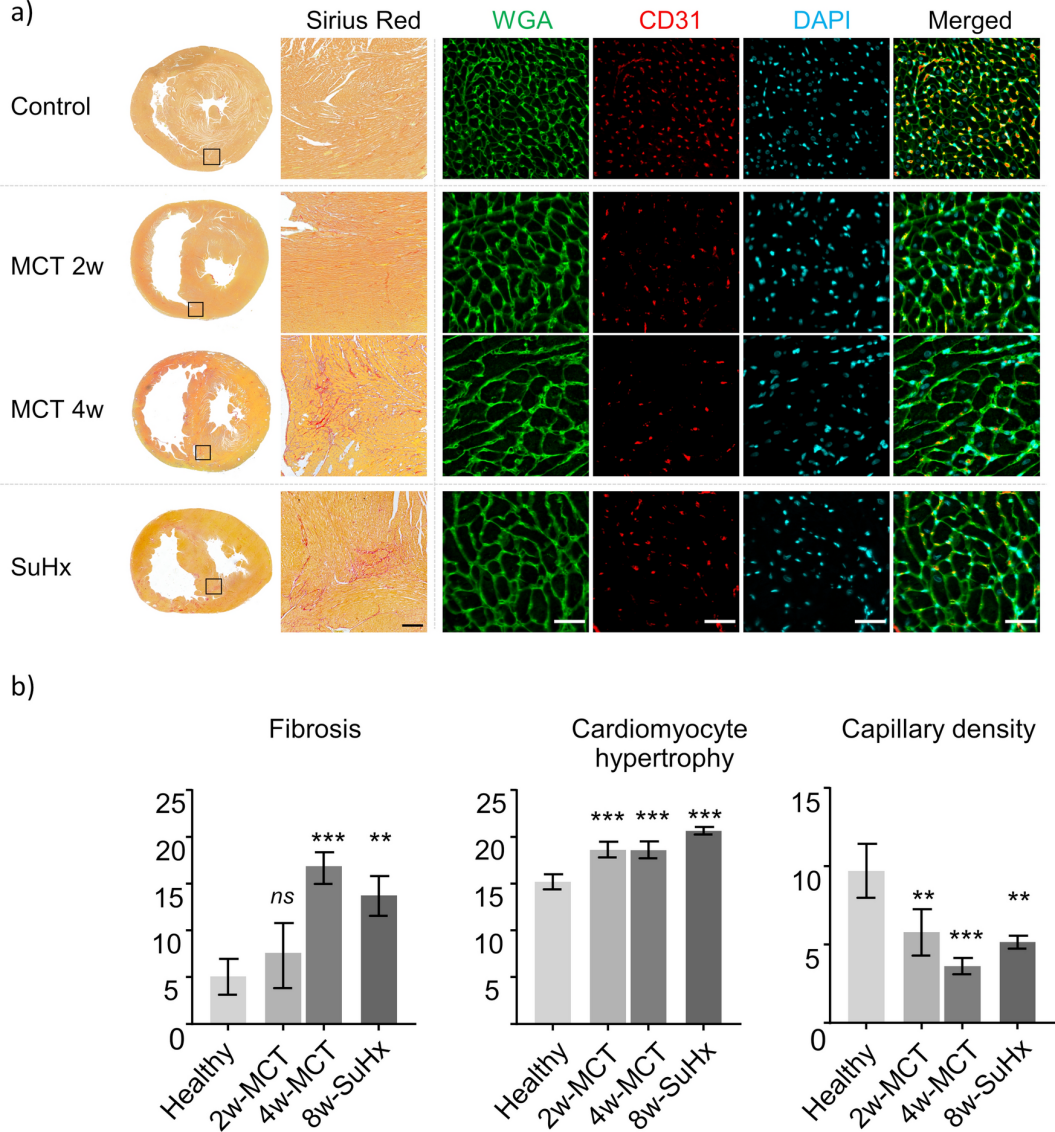
Histology and quantification of tissue damage parameters of MCT and SuHx rats. Representative picrosirius red stained whole hearts (selection box size: 1.35 mm × 1.35 mm) and magnified histology micrographs are displayed showing an example of fibrosis at RVIP (Picrosirius Red, scale bar 200 µm) for both MCT and SuHx rats. Significant increase in cardiomyocyte size was noted at 2-, 4-weeks MCT and 8-week SuHx (WGA, scale bar 100 µm). Reduced capillary density at 2-, 4-week MCT and 8-week SuHx (CD31, scale bar 100 µm) (values expressed as mean ± SD, *p < 0.05, **p < 0.01, ***p < 0.001, ****p < 0.0001, one-way ANOVA—comparison to healthy, Dunnett correction for multiple comparisons, n = 3 each group).

Magnified histology micrographs show an example of accumulation of fibrosis at RVIP and RV wall of MCT and SuHx rats. Picrosirius Red-stained sections demonstrated very mild interstitial fibrosis at 2-weeks MCT, followed by patchy fibrosis at 4-weeks MCT and 8-weeks SuHx. MCT and SuHx rats were observed to have higher myocyte diameter (WGA staining), reflecting myocyte hypertrophy, and reduced capillary density (CD31 straining) at RVIP (Fig. 5a). Myocyte hypertrophy occurred as early as 2-weeks MCT. At this stage, a moderate trend of reduced capillary density was observed. Profound loss of capillary density was present at 4-weeks MCT complemented by strong hypertrophy. As with 4-weeks MCT, at 8-weeks SuHx severe cardiomyocyte hypertrophy was present and colocalized with decreased capillary density (Fig. 5b).

### Female rat results

Compared to healthy controls, MCT (4-weeks) and SuHx (8-weeks) female rats presented significantly increased mean pulmonary arterial pressure (mPAP, MCT 45.3 ± 8.38 mmHg, SuHx 43.3 ± 15.3 mmHg vs. 17.2 ± 2.48 mmHg in controls) and increased RV systolic pressures at endpoints (Fig. S4 Supplement).

**Fig. 6:**
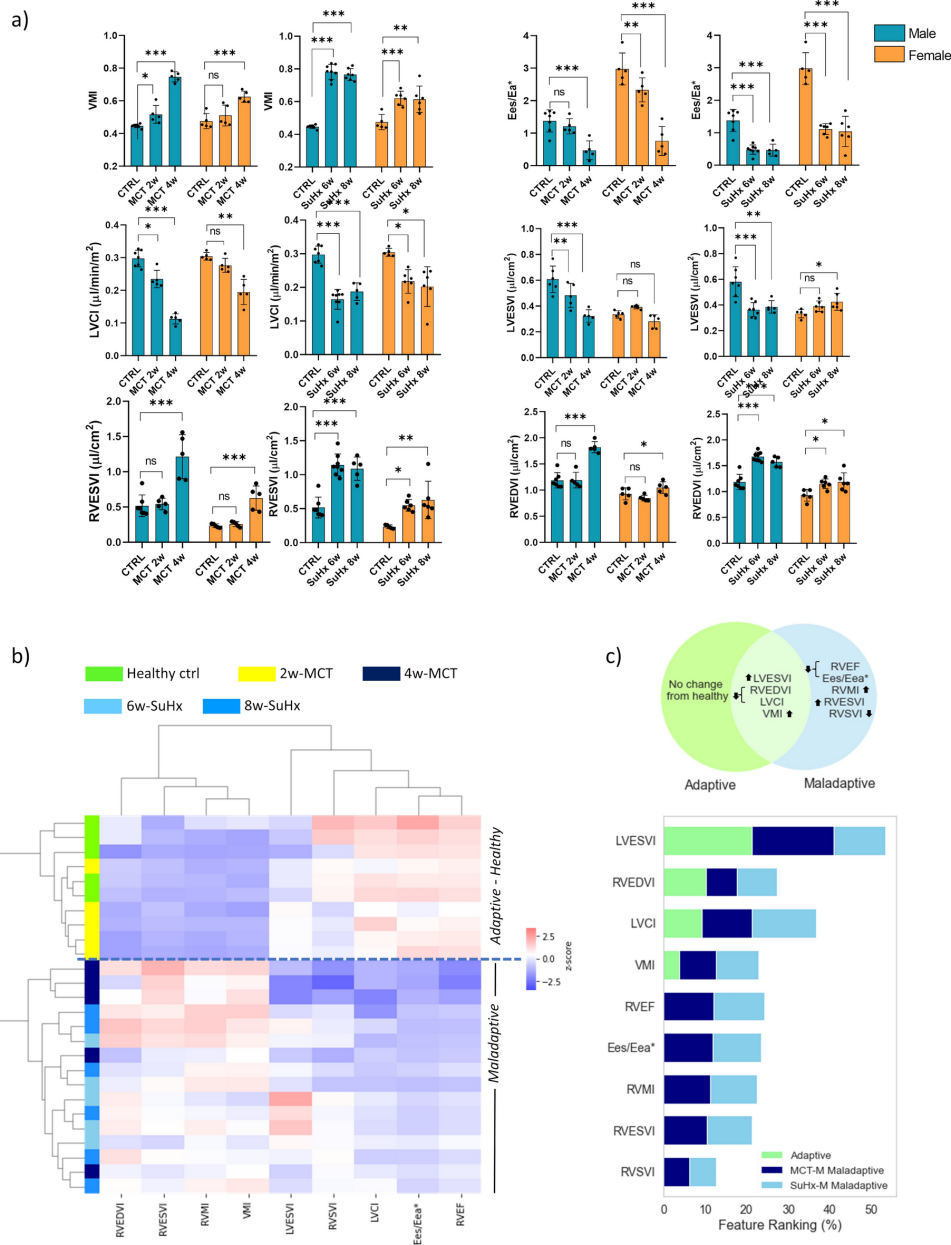
Longitudinal assessment of LV and RV in female rats. (**a**) MRI derived metrics to assess hypertrophy, dilatation, and systolic function of RV and LV for control, 2-, 4-week MCT and 6- and 8-week SuHx females (mean ± SD, *p < 0.05, **p < 0.01, *** p < 0.001, **** p < 0.0001, one-way ANOVA, Dunnett correction—comparison to healthy, n = 5 to 6 each group) compared with male counterparts. (**b**) Dendrogram and heat map of hierarchical clustering analysis. (**c**) Ranking of selected features based on their contribution to classify the adaptive/maladaptive MCT and SuHx females. Venn diagram highlighting common and distinct features of adaptive/maladaptive stages.

Female rats developed a comparable severity of RV phenotype to their male counterparts at end stage, although this occurred slightly later than in males. Maladaptive MCT and SuHx female rats demonstrated significant deterioration of functional markers (RVEF and coupling index). Yet among the MCT female rats, RVH and dilatation developed only at 4-week timepoint. Overall, MCT and SuHx female rats had less pronounced hypertrophy and dilatation as male counterparts (Fig. 6a).

The dendrogram and heat map from the hierarchical clustering showed that relevant parameters characterising the adaptive and maladaptive stage in female rats (Fig. 6b) were predominantly RV driven, comparable to male cohorts. End systolic dilatation of the LV was a particular feature of females, possibly a consequence of weaker RV dilatation (less LV compression). The ranking of parameters is presented in Fig. 6c, illustrating that dilatation of LV and RV are main features of the adaptive stage, followed by reduced LVCI and VMI increase (lesser extent compared to male counterparts). Ranking of maladaptive stage was characterised by LVESVI and RVEDVI increase, decline in RVEF and coupling index, increased RVMI, RVESVI and reduced RVSVI. Female MCT rats in our study, despite exhibiting significant fibrosis compared to control, showed markedly less fibrosis than male counterparts (Fig. S5 Supplement).

### Key molecular signatures involved in RV failure

**Fig. 7:**
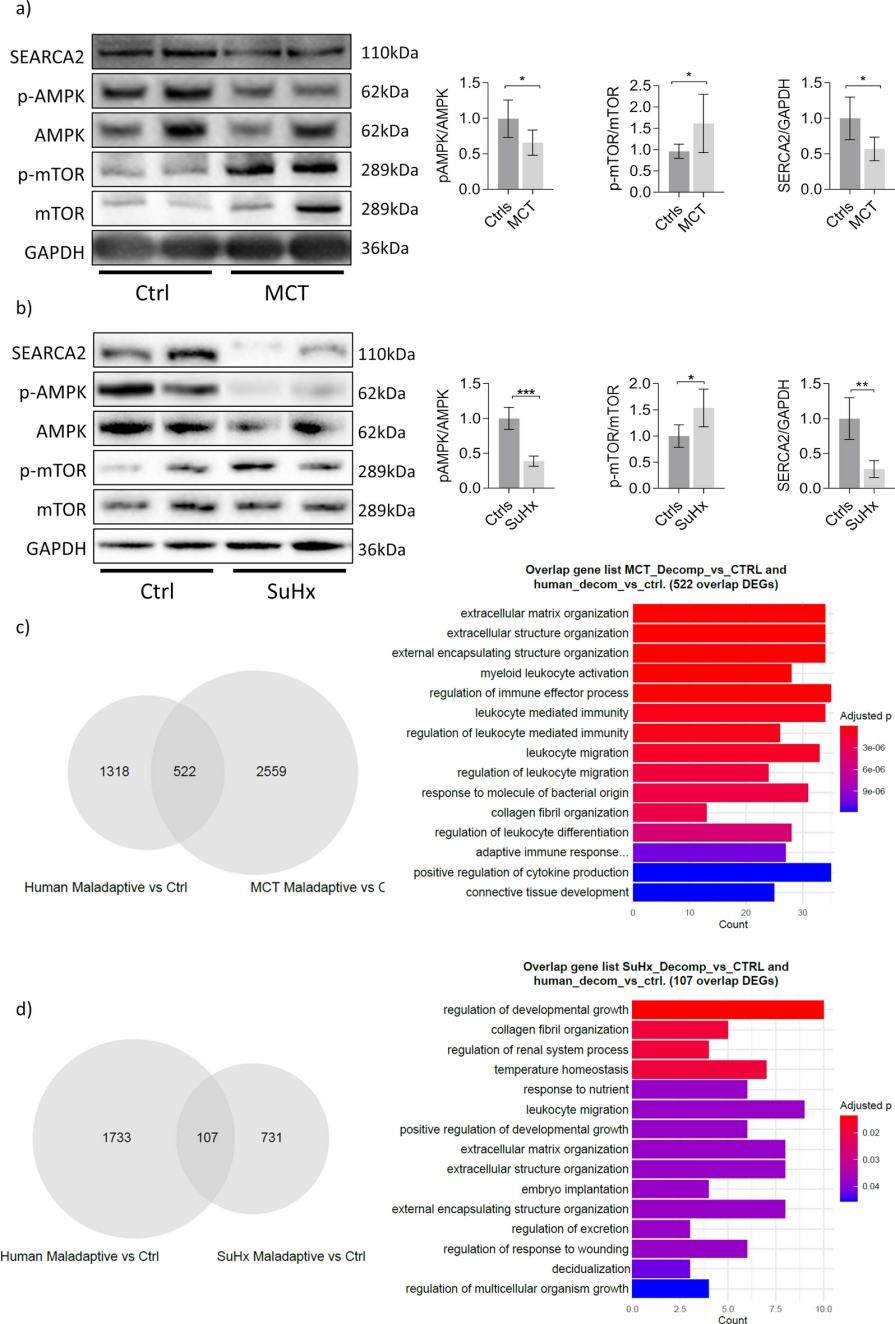
Expression of key molecular signatures involved in RV failure. (**a,b**) Representative western blots of RV tissue from MCT and SuHx rats, along with quantification. Key molecules shown: AMPKα and mTOR and their phosphates, and SERCA2 (mean ± SE, *p < 0.05, **p < 0.01, ***p < 0.001, ****p < 0.0001, n = 5 each group, t-test). The original, unprocessed blots are presented at the end of supplementary document (1) and (2). (**c**) Venn diagram highlighting common and distinct DEGs for decompensated MCT and human samples along with top significantly enriched pathways for the 522 common DEGs. (**d**) Venn diagram highlighting common and distinct DEGs for decompensated SuHx and human samples along with top significantly enriched pathways for the 107 common DEGs. Human RV tissues: control/normal (*n* = 13) and decompensated (*n* = 13).

Western blots were performed to characterise the molecular crosstalk between the key signalling (Fig. 7a). The phosphorylation of AMPK was downregulated in both MCT and SuHx rats compared to healthy controls (Fig. 7b). This change was accompanied by enhanced phosphorylation of mTOR. Furthermore, the expression of SERCA2 was significantly decreased in the MCT and SuHx rats. Comparing to human RV decompensated data set, the RNA-sequencing DEG results showed that MCT rats displayed 522 common genes (Fig. 7c) whereas SuHx demonstrated 107 genes (Fig. 7d). Focusing on the genes that were commonly regulated in all three profiles, we found that ECM and collagen fibrillar organisation were highly upregulated in both animal and human profiles along with responses to cytokines (Fig. 7c,d). These immune responses in decompensated MCT RV samples shared similarity to decompensated human RV samples, while growth differential factor was common in SuHx and RV human samples.

## Discussion

**Fig. 8:**
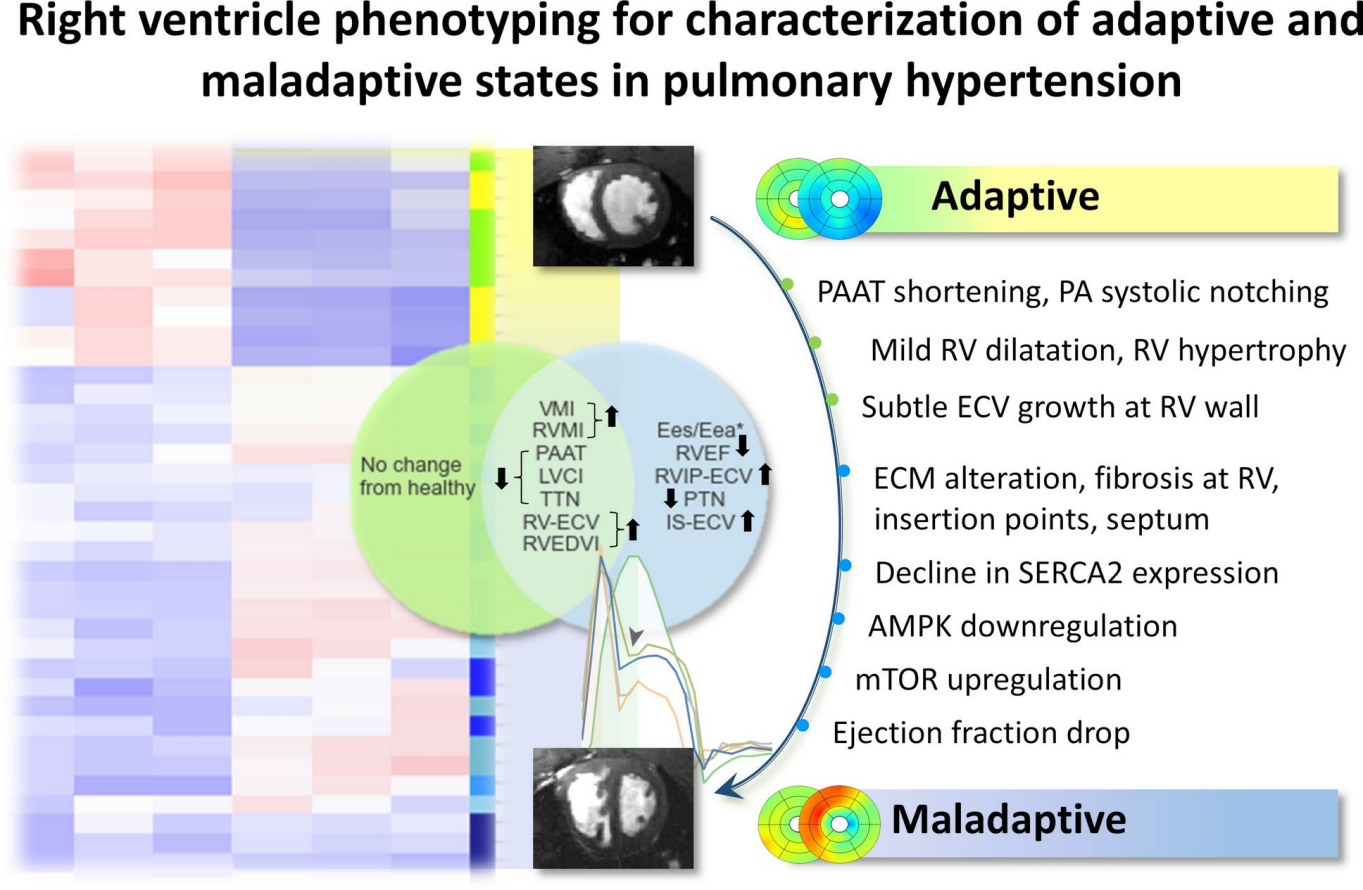
Central illustration summarising the main characteristics of adaptive and maladaptive stages of RV behaviour as identified in the MCT and SuHx animal models.

Our study established a unique cardiac platform based on MRI techniques of cine, flow sensitized and extracellular volume assessments to distinguish key characteristics of the RV remodelling process, non-invasively, in two of the most used experimental PH models. Cine-MRI evaluated RV dilation, hypertrophy, and systolic dysfunction, while flow MRI allowed measuring PAAT, TTN and PTN. Additionally, ECM organization was portrayed using ECV. Feature selection, combined with machine learning classification and ranking, determined the MR feature subset most relevant to cluster and characterise adaptive and maladaptive RV states, thereby establishing a standardised translational MRI platform for the PH research^5^. Haemodynamic alterations of PA blood flow ejection and wave reflection, mild RV dilatation, and progressive RVH with subtle extracellular volume growth in the RV wall were dominant features of the adaptive stage. Typical in vivo signatures of maladaptation included an increase in the extracellular matrix at RVIP and septum paralleled by profound RV dysfunction (reduced RVEF, and Ees/Eea*). The correlation network revealed distinct hub distributions related to RV deterioration, emphasising strong interdependences between hypertrophy and ECV as well as between PAAT, TTN, and RV-PA uncoupling. Histopathology, biochemical assays and RNA-sequencing validated the maladaptive characteristics of: (1) myocyte hypertrophy, capillary rarefaction, and fibrosis observed at maladaptive stage; (2) molecular mechanisms linking RV maladaptation with alterations of myocardial energetics and calcium handling; (3) increased transcription of ECM components in PH patients and both animal models suggesting aberrant ECM organisation. The main characteristics of adaptive and maladaptive RV states identified in this study are illustrated in Fig. 8.

The two used animal models are recognised in the PH research community and recapitulated some, if not all, typical signatures of PH development as seen in the PH clinic^9^. We show that the MCT rat model can sequentially replicate the adaptive and maladaptive RV states, exhibiting traits of adaptive phenotypes at 2-weeks after MCT induction, followed by signs of maladapted RV dysfunction and failure at 4-weeks. The SuHx model showed a longer span of constantly persistent, yet maladaptive process. The established MRI framework in this study reflected longitudinal RV remodelling at intervals of 2- to 4- weeks in MCT rats and 6- to 8-weeks in SuHx rats, offering suitable timeframes for evaluating efficacy of pharmaceutical reagents.

We were able to capture early physiological changes associated with RV adaptation. Flow MRI demonstrated that shortening of PAAT and TTN complemented by RVH were early phenotypic changes observed at adaptive stage, contributing 46% to the classification. These parameters played integrative role in predicting maladaptive RV responses in MCT and SuHx rats. PAAT is known to decline with increased PA pressure, indicating progressively worsening conditions of vasculature from increased stiffness/decreased compliance point of view^21^. In addition, the upstream inhibition of flow generates blood flow wave reflection, causing the characteristic notch seen in maladaptive stages. Our results affirm this, as not only PAAT declined with disease severity, but also TTN did. Earlier presence of the notch could indicate enhanced pressure burden on the RV caused by the reduced time for forward flow in the vessel^22^. These findings are supportive of the progressive pulmonary vasculature pathology associated with the SuHx model^15^. Notably, there were controversial views suggesting partial RV recovery in the SuHx model^23^. Our findings demonstrate continuous maladaptive remodelling of the RV upon re-normoxia exposure.

While the insertion points had been previously assessed using imaging, limited studies have (1) evaluated the extracellular volume status in the RV wall at adaptive stage (primarily because of RV myocardium thinness) and (2) demonstrated direct association with myocardial fibrosis. We demonstrated that an increased ECV at RV wall, along with RVH were relevant features of adaptive rats (11.9% contribution to classification), in keeping with the findings from the 2-weeks MCT picrosirius staining. Indeed, the integrity of extracellular matrix (ECM) appears crucial in preventing the pathological overstretch of RVH and RV dilation^24^. While increases in fibrillar collagen in ECM may initially help prevent ventricular dilatation, the loss of ECM integrity over time may lead to RV dysfunction and ultimately promote RVF. MCT rats exhibited severe septal ECV increase at 4-weeks, while SuHx had less pronounced enhancement primarily at the basal RV septal attachment and RVIP. This observation may indicate a severer maladaptive phenotype of the 4-week MCT rats, compared to the SuHx rats^25^. Indeed, RVH and septal displacement/bowing are recognised for imposing a high level of mechanical stress which exacerbates septal ECM disorganisation through myocardial fibre disarray and fibrosis development^24^. Since septal displacement/bowing indicates the onset of RV failure^26^, the increased ECV at RVIP may signal this event early, prior to septal augmentation, thus anticipating severe maladaptation. The picrosirius staining performed on the maladaptive RV tissue confirmed strong association between increases of extracellular volume and areas of pathological fibrosis. Analysis of microvessel density assessed by CD31 staining brought evidence of capillary density decrease, most marked at the RVIP at both adaptive and maladaptive stages. This reduction in capillary density may arise as a consequence of increased myocardial mass without proportionate growth of the microvascular bed.

The MRI indicators of adaptation and maladaptation outlined here hold significant promise for assessing therapeutic interventions aimed at improving RV function. While traditional indices such as RVEF, RV-PA (un)coupling, RVH and LVCI remain essential for evaluating functional responses to PH drugs, we show that ECV enhancement at RVIP and interventricular septum can serve as important integrative markers of disease severity^27^. RVIPs and subsequently the septum are the earliest and most intensely impacted by RV overloading^24^. For both animal models, decreases in ECV-IS and RV-IP ECV that may take place following therapeutic intervention, would indicate a favourable paradoxical IS motion response, signalling a diminished overload burden on the RV. In the absence of measurable ECV enhancement, an effective therapeutic intervention could be evaluated through indicators of RV and LV systolic functional improvement (RVEF, Ees-Eea*, LVCI), along with reduced RV dilatation and RVH. Ventricular hypertrophy and dilatation may not entirely recover and may co-exist with the noted growth of extracellular volume of the RV wall, as observed in the MCT and SuHx rats. In case the systolic function is restored to levels comparable to those of the healthy control group, these two indices would reflect traits of RV adaptation.

In addition to the MRI assessment, RNA-sequencing analysis of RV tissues collated through the in vivo experiment provided insight into molecular changes characterising the maladaptive stage. Similar to human RV decompensated state, our data indicated ECM alteration as a dominant factor in the development of RVF. ECM plays a pivotal role in the formation of both adaptive and maladaptive RV responses in PH through the development of myocardial fibrosis and the reorganisation of collagen fibrils^28^. We showed that increased transcription of ECM components occurs in the maladaptive RVs of PH patients and both animal models, thereby further validating the accuracy and translational value of the ECV results from this study.

There has been considerable interest raised by the PH taskforce in understanding the molecular mechanisms implicated in the RV myocardial energetics and calcium handling alterations seen in PH^5^. In response, our study showed that AMPK phosphorylation decreased in the maladaptive RV. AMPK is a critical energy sensor/regulator coordinating cellular metabolism with specific energy demands^29^ and its deficiency was noticed in the failing heart^18^. In our study, AMPK downregulation could be accounted for the enhancement seen on its downstream molecule mTOR. mTOR plays a fundamental role in promoting protein synthesis essential for hypertrophic growth of cardiomyocytes^30^. The upregulation of mTOR paralleled by AMPK deactivation seen at 4-week MCT and 8-week SuHx, emphasize the role the two molecules have in the regulation of maladaptive RV hypertrophy and RV failure in PH. Alterations in calcium handling had been shown to be involved in ventricular hypertrophy and dysfunction^31^. In line with this, we found reduced SERCA2 expression at 4-week MCT and 8-week SuHx. The function decline of SERCA2 found in the maladaptive RV suggests reduction of mitochondrial calcium dynamics and is strongly associated with the onset of RV systolic malfunction seen at this stage in vivo.

## Methods

### Animal models and experimental design

All animal experiments were conducted in accordance with the UK Home Office Animals (Scientific Procedures) Act 1986 and conform to the guidelines from Directive 2010/63/EU of the European Parliament on the protection of animals used for scientific purposes or the NIH Guide for the Care and Use of Laboratory Animals. All animal procedures were reviewed and approved by the Imperial College Animal Welfare and Ethical Review Body. The authors complied with the ARRIVE guidelines.

Adult male and female Sprague-Dawley rats (Charles River, UK) were used. PH was induced in two experimental models: (1) MCT model (subcutaneous MCT injection, 60 mg/kg; Sigma-Aldrich) and (2) SuHx model (subcutaneous SU5416 injection, 25 mg/kg; Tocris Bioscience), followed by 4-weeks exposure to normobaric hypoxia (FiO_2_ = 10%) and return to normoxia for 6 additional weeks. For the cardiac MRI experiment, anaesthesia was initiated with 3% isoflurane mixed with 100% oxygen in an induction chamber and maintained during the MRI procedure with 2% isoflurane mixed with 100% oxygen delivered via a nose cone. Rats were placed on a dedicated rat bed in prone position. Rat body temperature was maintained at ~ 36.5 °C using a warm water circulating heat mat. The electrocardiogram (ECG), respiration and body temperature were constantly monitored during the MRI scans (SA Instruments, Stony Brook, NY).

In vivo cardiac MRI was performed on males and females at baseline, 2- and 4-weeks MCT. SuHx rats were scanned at 4-, 6-, 8-, and 10-weeks for males and at 6- and 8-weeks for females. At the end of experiments, rats were euthanized with intraperitoneal injection of pentobarbital (150 mg/kg, i.p.) for tissue collection.

Tissue collection was performed at 4-weeks MCT and 8-weeks SuHx for histological and biochemical examination. To determine whether RV deterioration continued beyond 8-weeks SuHx, we performed cardiac MRI scanning at the 10-weeks SuHx timepoint (n = 5). We also collected tissue from 2-weeks MCT rats (n = 3) for validation of the in vivo results associated with the RV adaptive state. MCT and SuHx experimental data on males was collected from two independent examinations. The total rat number for males was: baseline (n = 12), 2-weeks MCT (n = 12), 4-weeks MCT (n = 9), 4-weeks SuHx (n = 7), 6-weeks SuHx (n = 15), 8-weeks SuHx (n = 10), 10-weeks SuHx (n = 10); for females: baseline (n = 5), 2-weeks MCT (n = 5), 4-weeks MCT (n = 5), 6-weeks SuHx (n = 6), 8-weeks SuHx (n = 6).

In this study, we selected the number of animals per group to match the minimum sample size required to achieve 85–95% statistical power. By implementing standardised imaging protocols and analysis pipelines to concurrently acquire and process functional, anatomical, and molecular information, our approach aligns with the 3Rs guidelines.

### Right heart catheterization

Selected rats from each independent experiment underwent catheterization to record mean pulmonary artery (PA) pressure (mPAP) and RV systolic pressure (RVSP) at baseline (n = 8), 4-weeks MCT (n = 6) and 8-weeks SuHx (n = 4) under anaesthesia (2.7 ml/kg i.p.) with Hypnorm (fentanyl/fluanisone): Hypnovel (midazolam): H2O = 1:1:2). Catheter ports were placed in the right atrium, right ventricle, and pulmonary artery. The pressure curves were recorded according to published guidelines^32^. The systemic systolic blood pressure (sSBP) was measured from the carotid artery.

### Cardiac MRI

#### Acquisition

Cardiac MRI was performed on a 9.4 T BioSpec scanner (Bruker BioSpin GmbH, Germany) equipped with a rat heart array receiver. Standardized cardiac planes were customized to match human protocols allowing reproducible planning of true short-axis orientation and full biventricular coverage. Vertical and horizontal long axis views (VLA and HLA) were captured bisecting the mitral valve and the apex by aligning the slices across the mid-ventricular transverse view, and along the VLA, respectively. Specific protocols were tailored, such as: (1) short axis 2D multi-stack cinematographic (Cine) MRI to assess global changes of RV and LV function, (2) phase contrast imaging (flow MRI) to measure the pulmonary artery acceleration time (PAAT) and systolic notch, (3) late gadolinium enhancement (*LGE*) and extracellular volume mapping (*ECV*) to locally quantify the distribution of pathological fibrosis at RV insertion points (RVIP), RV wall, and septum (IS)^33^.

Detailed description of acquisition methods, segmentation of ventricles and PA, list of parameters derived from cine, flow and ECV-MRI (Tables S1–S3 Supplement), analysis of flow-MRI, 26-segment model bullseye analysis of ECV (Fig. S1 Supplement) are found in the supplemental material.

#### Definitions

Ventricular hypertrophy (RVH, LVH) was defined by significant increase from healthy baseline values of: RV mass index (RVMI), LV mass index (LVMI) and ventricular mass index (VMI). Significant increase in end diastolic and end systolic volumes were taken as signatures of ventricular dilatation, while reduced LV end diastolic and end systolic volumes were referred to as LV compression. All MRI metrics were normalized to the body surface area (BSA)^34^ (Fig. S3, Supplement).

### Histology and biochemical analysis

Hearts of healthy, MCT and SuHx rats were excised and processed for histological and biochemical analysis. Fluorescent and immunohistochemical staining and quantification were performed to assess capillary density, myocyte size and collagen deposition. Western blotting assessed expression of AMPK, mTOR, p-mTOR and SERCA2 in the RV (supplemental methods). RNA sequencing was performed on rat RV tissues grouped into control (n = 8), maladaptive MCT (n = 6) and maladaptive SuHx (n = 3) (supplemental methods). RNA-sequencing data from a previous human study^28^ (performed on human RV tissues that were clinically classified by hemodynamic assessment and clinical symptoms into control/normal (*n* = 13) and decompensated (*n* = 13) RV states) was used for comparison of differentially expressed genes (DEG) results of all datasets. Control RV human tissues were obtained from patients with normal RV function for whom the previous medical history and/or the autopsy did not reveal cardiac or respiratory diseases. Decompensated RV human tissues were obtained from patients with end-stage PH who received a lung transplantation or who died with RV failure^28,35^. All experimental procedures for using human tissues conformed to the principles outlined in the Declaration of Helsinki. They were performed with the approval of Laval University and the Biosafety and Ethics committees of the University Institute of Cardiology and Respirology of Quebec (CER 20773, CER 20735, CER 21747). Human RV tissues were obtained from participants who had previously given written, informed consent.

### Statistical analyses

Statistical analysis was performed using GraphPad Prism 7 (GraphPad Software, San Diego, California). All results are presented as means with the corresponding standard deviations (± SD). One-way analysis of variance (ANOVA) was employed for multi-group analysis with Dunnett test for multiple comparison testing. Biochemical analyses were performed using Student t-test. A p-value of < 0.05 was taken to represent statistical significance.

#### MRI feature selection and statistical evaluation

A supervised statistical based approach was implemented for feature selection (FS)^36^. Specifically, the maximum relevance, minimum redundance (mRMR) algorithm was applied to select MRI parameters having highest correlation with MCT and SuHx classes (relevance) and least correlation between themselves (redundancy). The F-statistic calculated correlation with the class while Pearson correlation coefficient, the correlation between features. The least absolute shrinkage and selection (LASSO) regularisation algorithm was further applied to remove redundant features thereby enhancing the accuracy and interpretability of FS. All FS techniques were implemented using Python scikit-learn ML package (version 3.6)^37^. A random forest classifier was implemented to rank the selected features. The performance of FS was evaluated and validated by means of confusion matrix analysis and stratified four-fold cross-validation with train/test of 75/25 split ratio (scikit-learn, python 3.6). Area under the curve (AUC) assessed classifier performance respective to healthy/adaptive and maladaptive classes. The correlation network build on the MR driven parameters was implemented using the Python Networkx toolbox (version 3.9).

#### Sample size

Power analysis for one-tailed paired-samples t-test was performed^38^ to determine the minimum sample size needed to distinguish (1) the adaptive and maladaptive state from healthy and (2) maladaptive from adaptive groups. A statistical power of at least 85% and up to 95% was chosen with an alpha of 0.05.

## Conclusions

In conclusion, we characterised the functional landscape of RV progression from adaptation to failure in MCT and SuHx rats using non-invasive imaging. We highlighted the value of our proposed machine learning framework implemented from tailor-made cardiac MRI experiments to (1) detect early physiological changes of RV adaptation and (2) characterise and predict RV dysfunction in decompensated stage. These results can provide a foundation for establishing an imaging biomarker platform to evaluate treatment efficacy in preclinical studies by assessing hemodynamic impairments of PA, characterising the degree of ventricular dilatation/hypertrophy, evaluating the status of myocardial fibrosis and septal bowing, and identifying the eventual onset of systolic dysfunction. All methods outlined here are implemented on clinical scanners facilitating the translation of drug discovery studies into the clinical setting.

## Supplementary Information

Supplementary Information.

## Data Availability

All the RNAseq data of human and MCT rat RV analysed in this study are deposited in the Gene Expression Omnibus under accession number GSE240941^28^ link: https://www.ncbi.nlm.nih.gov/geo/query/acc.cgi?acc=GSE240941, while RNAseq data of SuHx rat RV are deposited in the Gene Expression Omnibus under the accession number GSE273007, link: https://www.ncbi.nlm.nih.gov/geo/query/acc.cgi?acc=GSE273007, respectively. All other data supporting the fundings in this study are available in the article or Supplemental.

## Abbreviations

MCT: Monocrotaline
SuHx: Sugen hypoxia
PA: Pulmonary artery
PH: Pulmonary hypertension
RV: Right ventricle
LV: Left ventricle
PH: Pulmonary hypertension
RVMI: ED end diastole
ES: End systole
EDVI: End diastolic volume index
ESVI: End systolic volume index
RVMI: Right ventricular mass index
VMI: Ventricular mass index
RVSVI: Right ventricular stroke volume index
LVSVI: Left ventricular stroke volume index
RVESVI: Right ventricular end systolic volume index
RVEF: Right ventricular ejection fraction
LVEF: Left ventricular ejection fraction
RVCI: Right ventricular cardiac index
LVCI: Left ventricular cardiac index
BW: Body weight
HR: Heart rate
BSA: Body surface area
FS: Feature selection
mRMR: Minimum redundance maximum relevance
Eea/Ees*: Surrogate of RV-PA uncoupling
PAAT: Pulmonary artery acceleration time
TTN: Time to notch
PTN: Peak to notch
T-ES: Time to end systole
PTES: Peak to end systole
ECV: Extracellular volume
RVIP: Right ventricular insertion points
IS: Interventricular septum
AMPK: AMP-activated protein kinase
mTOR: Mammalian target of rapamycin
SERCA2: Sarcoplasmic/endoplasmic reticulum Ca^2+^ ATPase

## References

[1] van de Veerdonk, M. C. et al. Progressive right ventricular dysfunction in patients with pulmonary arterial hypertension responding to therapy. *J. Am. Coll. Cardiol.* **58**, 2511–2519. 10.1016/j.jacc.2011.06.068 (2011). 22133851 10.1016/j.jacc.2011.06.068

[2] Haddad, F., Doyle, R., Murphy, D. J. & Hunt, S. A. Right ventricular function in cardiovascular disease, part II: Pathophysiology, clinical importance, and management of right ventricular failure. *Circulation* **117**, 1717–1731. 10.1161/CIRCULATIONAHA.107.653584 (2008). 18378625 10.1161/CIRCULATIONAHA.107.653584

[3] Dawes, T. J. W. et al. Machine learning of three-dimensional right ventricular motion enables outcome prediction in pulmonary hypertension: A cardiac MR imaging study. *Radiology* **283**, 381–390. 10.1148/radiol.2016161315 (2017). 28092203 10.1148/radiol.2016161315 PMC5398374

[4] Hoeper, M. M. et al. Mortality in pulmonary arterial hypertension: Prediction by the 2015 European pulmonary hypertension guidelines risk stratification model. *Eur. Respir. J.*10.1183/13993003.00740-2017 (2017). 28775047 10.1183/13993003.00740-2017

[5] Lahm, T. et al. Assessment of right ventricular function in the research setting: Knowledge gaps and pathways forward. An Official American Thoracic Society Research statement. *Am. J. Respir. Crit. Care Med.* **198**, e15–e43. 10.1164/rccm.201806-1160ST (2018). 30109950 10.1164/rccm.201806-1160ST PMC6835085

[6] Ho, S. Y. & Nihoyannopoulos, P. Anatomy, echocardiography, and normal right ventricular dimensions. *Heart* **92**(Suppl 1), i2-13. 10.1136/hrt.2005.077875 (2006). 16543598 10.1136/hrt.2005.077875 PMC1860731

[7] Voelkel, N. F. et al. Right ventricular function and failure: Report of a National Heart, Lung, and Blood Institute working group on cellular and molecular mechanisms of right heart failure. *Circulation* **114**, 1883–1891. 10.1161/CIRCULATIONAHA.106.632208 (2006). 17060398 10.1161/CIRCULATIONAHA.106.632208

[8] Xia, Y. et al. Super-resolution of cardiac MR cine imaging using conditional GANs and unsupervised transfer learning. *Med. Image Anal.* **71**, 102037. 10.1016/j.media.2021.102037 (2021). 33910110 10.1016/j.media.2021.102037

[9] Badagliacca, R. et al. Right ventricular remodeling in idiopathic pulmonary arterial hypertension: Adaptive versus maladaptive morphology. *J. Heart Lung Transpl.* **34**, 395–403. 10.1016/j.healun.2014.11.002 (2015). 10.1016/j.healun.2014.11.002 25499139

[10] van Wolferen, S. A. et al. Prognostic value of right ventricular mass, volume, and function in idiopathic pulmonary arterial hypertension. *Eur. Heart J.* **28**, 1250–1257. 10.1093/eurheartj/ehl477 (2007). 17242010 10.1093/eurheartj/ehl477

[11] Kiely, D. G. et al. EXPRESS: Statement on imaging and pulmonary hypertension from the Pulmonary Vascular Research Institute (PVRI). *Pulm. Circ.* **9**, 2045894019841990. 10.1177/2045894019841990 (2019). 30880632 10.1177/2045894019841990 PMC6732869

[12] Cerne, J. W. et al. Evaluation of pulmonary hypertension using 4D flow MRI. *J. Magn. Reson. Imaging* **56**, 234–245. 10.1002/jmri.27967 (2022). 34694050 10.1002/jmri.27967

[13] Alabed, S. et al. Myocardial T1-mapping and extracellular volume in pulmonary arterial hypertension: A systematic review and meta-analysis. *Magn. Reson. Imaging* **79**, 66–75. 10.1016/j.mri.2021.03.011 (2021). 33745961 10.1016/j.mri.2021.03.011

[14] Bueno-Beti, C., Sassi, Y., Hajjar, R. J. & Hadri, L. Pulmonary artery hypertension model in rats by monocrotaline administration. *Methods Mol. Biol.* **1816**, 233–241. 10.1007/978-1-4939-8597-5_18 (2018). 29987824 10.1007/978-1-4939-8597-5_18

[15] de Raaf, M. A. et al. SuHx rat model: Partly reversible pulmonary hypertension and progressive intima obstruction. *Eur. Respir. .J* **44**, 160–168. 10.1183/09031936.00204813 (2014). 24791833 10.1183/09031936.00204813

[16] Stenmark, K. R., Meyrick, B., Galie, N., Mooi, W. J. & McMurtry, I. F. Animal models of pulmonary arterial hypertension: The hope for etiological discovery and pharmacological cure. *Am. J. Physiol. Lung Cell Mol. Physiol.* **297**, L1013-1032. 10.1152/ajplung.00217.2009 (2009). 19748998 10.1152/ajplung.00217.2009

[17] Sanz, J. et al. Right ventriculo-arterial coupling in pulmonary hypertension: A magnetic resonance study. *Heart* **98**, 238–243. 10.1136/heartjnl-2011-300462 (2012). 21917658 10.1136/heartjnl-2011-300462

[18] Vonk-Noordegraaf, A. et al. Right heart adaptation to pulmonary arterial hypertension: Physiology and pathobiology. *J. Am. Coll. Cardiol.* **62**, D22-33. 10.1016/j.jacc.2013.10.027 (2013). 24355638 10.1016/j.jacc.2013.10.027

[19] Llucia-Valldeperas, A., de Man, F. S. & Bogaard, H. J. Adaptation and maladaptation of the right ventricle in pulmonary vascular diseases. *Clin. Chest Med.* **42**, 179–194. 10.1016/j.ccm.2020.11.010 (2021). 33541611 10.1016/j.ccm.2020.11.010

[20] Bekedam, F. T., Goumans, M. J., Bogaard, H. J., de Man, F. S. & Llucia-Valldeperas, A. Molecular mechanisms and targets of right ventricular fibrosis in pulmonary hypertension. *Pharmacol. Ther.* **244**, 108389. 10.1016/j.pharmthera.2023.108389 (2023). 36940790 10.1016/j.pharmthera.2023.108389

[21] Levy, P. T. et al. Pulmonary artery acceleration time provides a reliable estimate of invasive pulmonary hemodynamics in children. *J. Am. Soc. Echocardiogr.* **29**, 1056–1065. 10.1016/j.echo.2016.08.013 (2016). 27641101 10.1016/j.echo.2016.08.013 PMC5408579

[22] Rolf, A. et al. Right ventricular adaptation to pulmonary pressure load in patients with chronic thromboembolic pulmonary hypertension before and after successful pulmonary endarterectomy—A cardiovascular magnetic resonance study. *J. Cardiovasc. Magn. Reson.* **16**, 96. 10.1186/s12968-014-0096-7 (2014). 25475583 10.1186/s12968-014-0096-7 PMC4256924

[23] Toba, M. et al. Temporal hemodynamic and histological progression in Sugen5416/hypoxia/normoxia-exposed pulmonary arterial hypertensive rats. *Am. J. Physiol. Heart Circ. Physiol.* **306**, H243-250. 10.1152/ajpheart.00728.2013 (2014). 24240870 10.1152/ajpheart.00728.2013 PMC3920132

[24] Andersen, S., Nielsen-Kudsk, J. E., Vonk Noordegraaf, A. & de Man, F. S. Right ventricular fibrosis. *Circulation* **139**, 269–285. 10.1161/CIRCULATIONAHA.118.035326 (2019). 30615500 10.1161/CIRCULATIONAHA.118.035326

[25] Becker, M. A. J. et al. Correlation between septal midwall late gadolinium enhancement on CMR and conduction delay on ECG in patients with nonischemic dilated cardiomyopathy. *Int. J. Cardiol. Heart Vasc.* **26**, 100474. 10.1016/j.ijcha.2020.100474 (2020). 32021905 10.1016/j.ijcha.2020.100474 PMC6994302

[26] Fakhri, A. A., Hughes-Doichev, R. A., Biederman, R. W. & Murali, S. Imaging in the evaluation of pulmonary artery hemodynamics and right ventricular structure and function. *Heart Fail Clin.* **8**, 353–372. 10.1016/j.hfc.2012.04.004 (2012). 22748899 10.1016/j.hfc.2012.04.004

[27] Swift, A. J. et al. LGE patterns in pulmonary hypertension do not impact overall mortality. *JACC Cardiovasc. Imaging* **7**, 1209–1217. 10.1016/j.jcmg.2014.08.014 (2014). 25496540 10.1016/j.jcmg.2014.08.014

[28] Fatemeh Khassafi, P. C. et al. Transcriptional profiling unveils molecular subgroups of adaptive and maladaptive right ventricular remodeling in pulmonary hypertension. *Nat. Cardiovasc. Res.* **2**, 917–936 (2023). 39196250 10.1038/s44161-023-00338-3 PMC11358157

[29] Zhao, Q., Song, P. & Zou, M. H. AMPK and pulmonary hypertension: Crossroads between vasoconstriction and vascular remodeling. *Front. Cell Dev. Biol.* **9**, 691585. 10.3389/fcell.2021.691585 (2021). 34169079 10.3389/fcell.2021.691585 PMC8217619

[30] Xu, L. & Brink, M. mTOR, cardiomyocytes and inflammation in cardiac hypertrophy. *Biochimica et Biophysica Acta (BBA) Mol. Cell Res.* **1863**, 1894–1903. 10.1016/j.bbamcr.2016.01.003 (2016). 10.1016/j.bbamcr.2016.01.003 26775585

[31] Toya, T. et al. Impact of oxidative posttranslational modifications of SERCA2 on heart failure exacerbation in young patients with non-ischemic cardiomyopathy: A pilot study. *Int. J. Cardiol. Heart Vasc.* **26**, 100437. 10.1016/j.ijcha.2019.100437 (2020). 31763443 10.1016/j.ijcha.2019.100437 PMC6864308

[32] Rosenkranz, S. & Preston, I. R. Right heart catheterisation: Best practice and pitfalls in pulmonary hypertension. *Eur. Respir. Rev.* **24**, 642–652. 10.1183/16000617.0062-2015 (2015). 26621978 10.1183/16000617.0062-2015 PMC9487613

[33] Kim, P. K. et al. Serial T1 mapping of right ventricle in pulmonary hypertension: Comparison with histology in an animal study. *J. Cardiovasc. Magn. Reson.* **23**, 64. 10.1186/s12968-021-00755-y (2021). 34039372 10.1186/s12968-021-00755-y PMC8157452

[34] Gouma, E. et al. A simple procedure for estimation of total body surface area and determination of a new value of Meeh’s constant in rats. *Lab Anim.* **46**, 40–45. 10.1258/la.2011.011021 (2012). 22008848 10.1258/la.2011.011021

[35] Omura, J. et al. Identification of long noncoding RNA H19 as a new biomarker and therapeutic target in right ventricular failure in pulmonary arterial hypertension. *Circulation* **142**, 1464–1484. 10.1161/CIRCULATIONAHA.120.047626 (2020). 32698630 10.1161/CIRCULATIONAHA.120.047626

[36] Anaraki, J. R. & Usefi, H. A comparative study of feature selection methods on genomic datasets. *Comp. Med. Sy.*10.1109/Cbms.2019.00097 (2019).

[37] Pedregosa, F. et al. Scikit-learn: Machine learning in python. *J. Mach. Learn Res.* **12**, 2825–2830 (2011).

[38] Faul, F., Erdfelder, E., Lang, A. G. & Buchner, A. G*Power 3: A flexible statistical power analysis program for the social, behavioral, and biomedical sciences. *Behav. Res. Methods* **39**, 175–191. 10.3758/bf03193146 (2007). 17695343 10.3758/bf03193146

