# Supplementary material for: Deep phenotyping the right ventricle to establish translational MRI biomarkers for characterization of adaptive and maladaptive states in pulmonary hypertension
Source: Sci Rep. 2024 Nov 30;14:29774. doi: 10.1038/s41598-024-79029-3 (PMC11608234; doi:10.1038/s41598-024-79029-3)

**Deep phenotyping the right ventricle to establish translational MRI biomarkers for characterization of adaptive and maladaptive states in pulmonary hypertension**

Nicoleta Baxan^1,2^, Lin Zhao^1^, Ali Ashek^1,2^, Marili Niglas^1^, Dingyi Wang^1^, Fatemeh Khassafi^3,4^, Farah Sabrin^2^, Olivier Dubois^2^, Chien-Nien Chen^1^, Soni Savai Pullamsetti^3,4^, Martin Wilkins^1^, Lan Zhao^1,2^*

**Supplementary material**

**Methods**

**Cardiac MRI**

Data was acquired with Paravision 6.0.1. All data were prospectively double respiratory- and cardiac (R wave)- triggered. Information of MR acquisition is detailed below:

*Cine-MRI*: cine short axis multi-stack was acquired with a gradient echo spoiled (FLASH) sequence with: repetition time (TR)=RR interval/number of frames (∼6.2 ms for ∼27 frames), TR_effective_=RR interval, echo time (TE)=2.2 ms, flip angle 18°, slice thickness ≤1.4 mm, 14 to 15 slices, acquisition matrix (188×188), field of view (38×38) mm^2^, spatial in-plane resolution (202 ×202) μm^2^, total scan time 18 min. with triggering. Frames covered a full cardiac cycle.

*Flow-MRI*: 2D phase-contrast MRI was acquired on a single slice placed midway between the level of the pulmonary valve and the bifurcation of the branch of pulmonary arteries. The slice was aligned to be perpendicular to the course of the vessel. To ensure optimal image angulation, two double-oblique cine MR views oriented along the main axis of the pulmonary trunk were used for planning; velocity encoding was adjusted to avoid aliasing (VENC ranging from 100 to 140 cm/s). Additional parameters were: temporal resolution 6 ms, TE=2.6 ms, flip angle=25°, matrix size (128 x 128), in-plane spatial resolution (313 x 313) µm^2^, slice thickness 1.5 mm, 2 averages, scan time 4 min. with triggering. Frames covered a full cardiac cycle.

*LGE*: Short axis LGE images were acquired 35 to 40 min after contrast agent injection (0.5 mmol/kg Gadovist) using a multi-slice IR-FLASH sequence with single inversion time (TI) and 90° flip angle. TI was selected to null the healthy myocardium. The TR between each phase encoding pulse was 3.6 ms, TE=1.39 ms, slice thickness 1 mm, FOV (36 × 36) mm^2^, acquisition matrix (180 × 180), in plane resolution (200×200) μm^2^, with 10 to 11 slices typically needed to fully cover the IVS from apex to base. Scan time was 2 min 30 s per slice including triggering.

*T_1_ mapping and ECV*: T_1_ mapping was performed before and 20 to 35 min after contrast agent injection. T_1_ maps were acquired in true short axis orientation at three equidistant locations as per AHA recommendations: basal, mid-ventricular and apical level. A gradient echo-based look-locker inversion recovery sequence was employed with an adiabatic global inversion followed by 20 inversion times (TI). All inversions were R wave triggered to allow data to be acquired at the same part of the cardiac cycle (end diastole). The TI points were restricted to multiples of the R-R interval (R-R ranging from 160 ms to 200 ms). Additional parameters were: inversion repetition time: 6 s (to allow full relaxation between inversions), TR = 4.5 ms, TE = 2.1 ms, flip angle 7°, slice thickness 1.5 mm, 3 slices, field of view (36 × 36) mm^2^, spatial in plane resolution (214×214) μm^2^, with 4 min. of scan time for each slice.

*Ventricular and pulmonary artery segmentation*: Epicardial and endocardial contours were traced manually to define the ventricular wall. IS was considered part of the LV. To avoid signal contamination from the blood pool, the outer and innermost 5% of the myocardial wall were excluded. Papillary muscles and trabeculations were part of the blood pool. All MRI metrics were normalized to the body surface area (BSA) (reference 34 in main manuscript). The main PA lumen was delineated at end systolic PC-MRI magnitude images, propagated automatically to subsequent cardiac frames and co-registered to corresponding velocity encoded images.

**Histology**

Following imaging, the hearts were excised and immediately fixed in 4% neutral formalin solution for 24h followed by transferring to 70% ethanol. Formalin fixed whole heart samples were processed for paraffin embedding and cross-sectioned to obtain 5 μm-thick sections at the midventricular level. Sections were deparaffinized, rehydrated and processed for routine haematoxylin/eosin and histochemical staining. Quantitative analysis was carried out independently by two blinded investigators (L.Z. and A.A.). Briefly, the slides were incubated with a 0.1% Sirius Red solution dissolved in aqueous saturated picric acid for 1 hour, washed in acidified water (0.5% hydrogen chloride), dehydrated and mounted with DPX Mounting.

For double immunofluorescence, sections were incubated with CD 31 (CD31; 1/100, Abcam), and Alexa 488 conjugated WGA (1/1000; ThermoFischer), detected with secondary antibodies, Alexa 568 anti-rabbit (1/1000; Invitrogen), with mounting solution containing 4′,6-diamidino-2-phenylindole (Vector Laboratories). Images were obtained with a Zeiss Axio Observer microscope (Carl Zeiss Microscopy, Munich, Germany).

For quantification of fibrosis all picrosirius red stained images were taken under polarized light and analysed using ImageJ (National Institutes of Health, Bethesda, MD). The percentage of fibrosis compared to total area of tissue within each image under a microscopy magnification of ×20. For each sample a minimum 10 snaps under polarized light were taken to cover the RV insertion points. WGA and CD31 stained images were used to measure myocyte size and capillary number respectively. Both anterior and inferior RV insertion points were scanned at 20x magnification from at least 4 whole heart sections from each group. These images were analysed using Image J based macro to identify minimum feret for cardiomyocyte diameter and number of CD31 positive micro-capillary for density measurements.

**Western blotting**

For protein extraction, RV tissues were homogenised in radioimmunoprecipitation lysis buffer (Sigma) using an electronic homogenizer (Kinematica, Switzerland). Protein samples were separated by electrophoresis in 4-12% NuPAGE® Bis-Tris gels (Invitrogen) and then transferred to nitrocellulose membranes (Bio-Rad). After blocking, the membranes were probed with the following antibodies: *anti-AMPK* (Cell Signaling Technology, #2532S, 1:1000); *anti-phospho-AMPK* (Thr172) (Cell Signaling Technology, #2535S, 1:1000); *anti-mTOR* (Cell Signaling Technology, #2983S, 1:1000); and *anti-phospho-mTOR* (Ser2448) (Cell Signaling Technology, #5536S, 1:1000); *anti-SERCA2* (Abcam, ab2861, 1:1000); *anti- UCP2* (Abcam, ab67241, 1:1000); *anti-GAPDH* (Sigma-Aldrich, G9545, 1:2000). Horseradish peroxidase (HRP) conjugated secondary antibody was incubated and then the chemiluminescent HRP substrate (Millipore) was applied. Blot signals were detected using a chemiluminescence detection system (Bio-Rad). The relative intensity of the blots was analysed using ImageJ software.

**Total RNA isolation and purification**

Total RNA was isolated from frozen RV tissues of the experimental models using the RNeasy Mini Kit (Qiagen). The tissues (no more than 30mg) were homogenized in 500µL Buffer RLT (containing 0.01% ß-mercaptoethanol). The homogenate was then mixed with 1 volume of 70% ethanol and was transferred to RNeasy Mini spin column and centrifuged. After this step, the on-column DNase digestion was performed following the manufacture instruction. Following the DNase digesting steps, the column was washed with 700µL Buffer RW1 for once and 500 µL Buffer RPE for twice sequentially. Then the spin column was transferred to RNA collection tubes, and the RNA was eluted in 30µL RNase-free water. RNA concentration and quality were estimated using a NanoDrop Spectrophotometer.

The absorbance at 260nm (A260), 280nm (A280) and 230(A230) were used to assess the quality of the RNA samples. The peak of the absorption spectrum of nucleotides is at A260. The RNA quality is accepted when both the A260/A280 ratio was around 2.0 and the A260/A280 ratio was higher than the A260/A280 ratio.

**Library preparation and sequencing**

The total RNA was submitted to Imperial Genomics Facility for library preparation and sequencing. The sequencing library was constructed from the total RNA using the TruSeq RNA Sample Preparation Kit (Illumina) following the product manual. Then the library was subjected to Illumina HiSeq 4000 platform and was sequenced with the read depth of 25 million reads per sample. The raw sequencing data were archived in FASTQ files. The FASTQ files, along with the quality control reports, were returned to us for analysis.

**Analysis**

**Cardiac MRI analysis**

Cardiac MR images were converted in Dicom and were analysed in Segment (Segment V2.2 R6190) and ArtFun (Inserm, Paris, France). Image segmentation and data analysis were performed independently by three experienced imaging researchers in a blinded manner (N.B., L.Z., M.N.).

*Cine-MRI*: Epicardial and endocardial contours were traced manually to define the ventricular wall at end diastole (ED) and end systole (ES). Stroke volume (SV) was defined as the difference between ED volume (EDV) and ES volume (ESV); SV = EDV – ESV. Cardiac output (CO) was derived from the following calculation: CO = HR * SV / 1000, units are ml/min. The EF was defined as EF = SV / EDV * 100%. Ventricular mass was calculated by multiplying the specific myocardial density (1.05 g /ml) with the ED myocardial volume. All indexed parameters were matched to the body surface area (BSA). This was calculated with Meeh’s formula: BSA = k * w ^2/3^ / 10000, where k is a constant 9.83 and *w* is body weight in grams, resulting BSA is in cm^2^.

*Flow-MRI analysis*: PAAT was defined as the time interval from the onset of blood flow in systole to peak systolic flow. The point where the highest velocity/flow was achieved was the peak, and an abnormal mid-systolic deceleration from the peak was defined as the notch. The dicrotic notch was defined as the systolic deceleration occurring in the pulmonary flow time profile. Time to notch (TTN) was calculated as the duration from the onset of blood flow in systole to notch. Peak to notch (PTN) as the duration from the peak velocity to notch. Other derived parameters: slope (slope of the curve from the onset of blood flow to peak flow), time to end systolic flow (T-ES), peak to end systolic flow (PTES). The notch was a consistent finding in all animals, yet in control rodents the notch was subtle and almost non-existent. The data was normally distributed.

*T_1_ mapping*: Pixel-wise T_1_ curve fitting was performed on pre- and post Gd T_1_ maps using a non-linear least square three-parameter fit which accounted for Look-Locker correction. The inversion points acquired during respiratory motion were manually removed. The retrieved pre- and post T_1_ maps were automatically aligned and the ECV maps were generated (ref [18](#_ENREF_22) in main manuscript). A standardised segmentation model of LV and RV was established to assess ECV regional changes. To accomplish this, the 16-segment American Heart Association (AHA) model of the human left ventricle was customised to include the right ventricle with 10-segments selected as in figure below:


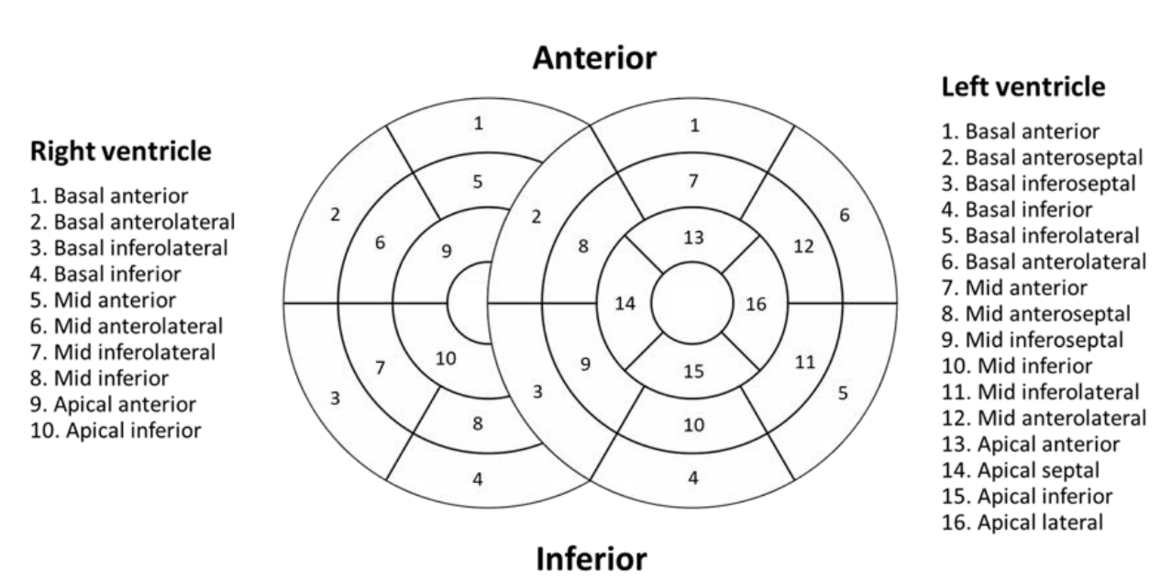


**Figure S1: Representative view of the 26-segment model used to assess ECV alterations regionally in both RV and LV.**

*RV-PA coupling*: In vivo, ventricular contractility for afterload is defined by the ratio between maximal elastance (E_max_) and effective arterial elastance (Ea). We used a simplified approach by measuring the ratio of volumes exclusively derived from CMR: E_max_/E_a_=SV/ESV (ref 17 in main manuscript).

**RNA Sequencing analysis**

The analysis steps included: (i) mapping the sequencing reads to the genome; (ii) quantification of the reads/abundance; (iii) normalization of the expression matrix; (iv) computing the differential expressing genes (DEGs); (v) functional analysis of the DEGs.

Sequencing data (FASTQ files) were subjected to Salmon (version 0.14.1) for reads mapping and counting. The transcriptome index used for reads mapping was built with the mice genome, transcriptome and annotation files (GRCm38.p6). DEGs between groups were computed using the built-in Wald statistic of the DESeq2 R package (version 1.24.0). Benjamini-Hochberg procedure was applied to control the false discovery rate (FDR) and to offer the *p* values with adjustment. The adjusted *p* value < 0.01 and the fold change > 2 were set as the criteria of DEGs. The annotation of the DEGs and the pathway enrichment were performed using clusterProfiler R package (version 3.12.0).

RNA sequencing data were processed with the R packages as mentioned above, and the statistics were concluded using the built-in algorithms. *** stands for *p* < 0.001; ** stands for *p* < 0.01; * stands for *p* < 0.05; *ns* stands for no significance.

**Results**

**Right heart catheterization**

Mean pulmonary artery (PA) pressure (mPAP) and RV systolic pressure (RVSP) at baseline, 4-weeks MCT and 8-weeks SuHx recorded under isoflurane anaesthesia for male rats are presented below (Fig S2).


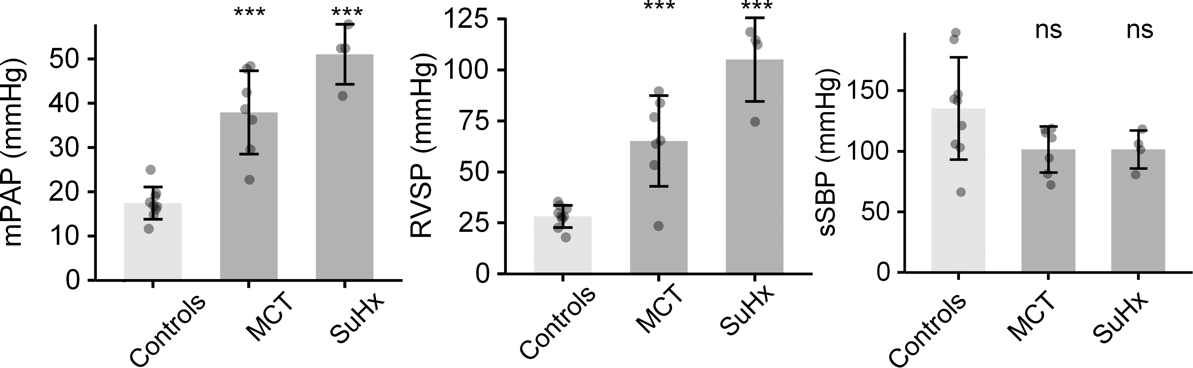


**Figure S2: Hemodynamic measurements from male MCT and SuHx rats compared to healthy controls.** Quantitative measurements of pulmonary artery mean pressure (mPAP), right ventricular systolic pressure (RVSP) and systolic blood pressure (sSBP): controls (n=9), MCT (n=7), SuHx (n=4). Significance values were: * p<0.05; ** p<0.01, *** p<0.001, one-way ANOVA, Dunnett correction, comparison to control group.

Body weight did increase along the course of the experiment, therefore, to account for this variation, all MRI metrics were normalized to the body surface area (BSA). The heart rate mildly decreased in both MCT and SuHx rats. Body weight, BSA and heart rate variation plotted at each experimental timepoint is summarised in Figure S3.

**
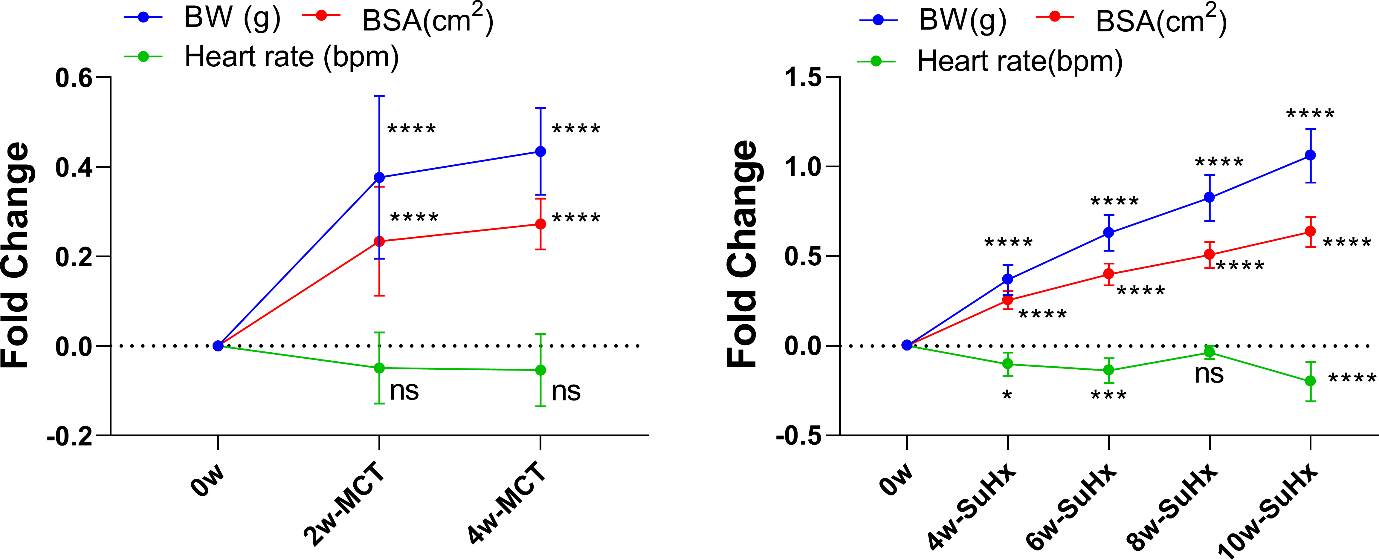
**

**Figure S3: Variation of body weight (BW), body surface area (BSA) and heart rate (HR) of the two animal models at all experimental timepoints.** Animal numbers: 0w (n=12), 2-weeks MCT (n=12), 4-weeks MCT (n=9), 4-weeks SuHx (n=7), 6-weeks SuHx (n=15), 8-weeks SuHx (n=10), 10-weeks SuHx (n=10), one-way ANOVA - comparison to controls, Dunnett correction for multiple variables.* p<0.05; ** p<0.01, *** p<0.001 comparison to control group.

Mean pulmonary artery (PA) pressure (mPAP) and RV systolic pressure (RVSP) at baseline, 4-weeks MCT and 8-weeks SuHx recorded under isoflurane anaesthesia for female rats are presented below (Fig S4).


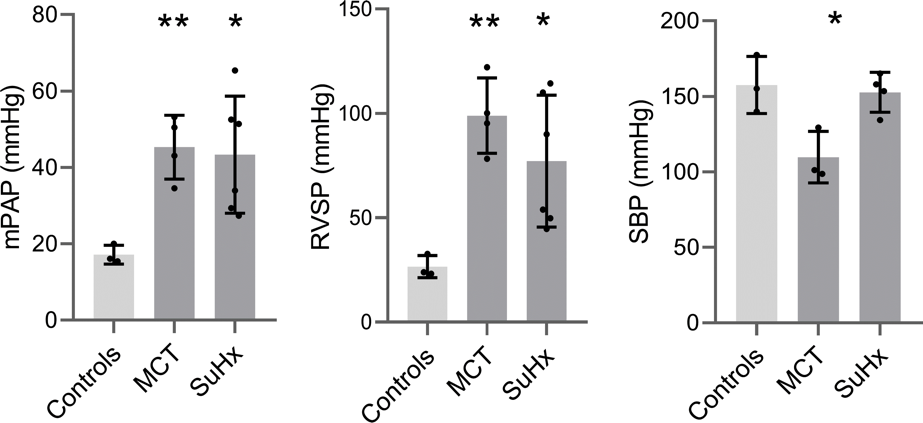


**Figure S4: Hemodynamic measurements from female MCT and SuHx rats compared to healthy controls.** Quantitative measurements of pulmonary artery mean pressure (mPAP): controls (n=3), MCT (n=4), SuHx (n=6), right ventricular systolic pressure (RVSP): controls (n=3), MCT (n=4), SuHx (n=6) and systolic blood pressure (sSBP) controls (n=3), MCT (n=3), SuHx (n=4), one-way ANOVA - comparison to controls, Dunnett correction for multiple variables. * p<0.05; ** p<0.01, *** p<0.001 when compared to control group.

**Histological assessment female MCT rats: Picrosirius red, WGA and CD31 results**

The female MCT animal models displayed significantly increased cardiomyocyte hypertrophy (Control: 13.5 ± 2.35 μm and MCT: 17.7 ± 2.27 μm) and loss in capillary density (Control: 3.1 ± 1.57% and MCT: 0.8 ± 0.14% from MCT; **Figure S5**). Elevated collagen deposition was noted in the MCT samples (control: 1.5 ± 0.44% and MCT: 4.6 ± 1.16%). Interestingly, the fibrosis development was more substantial in the male MCT rodents compared to female counterparts.


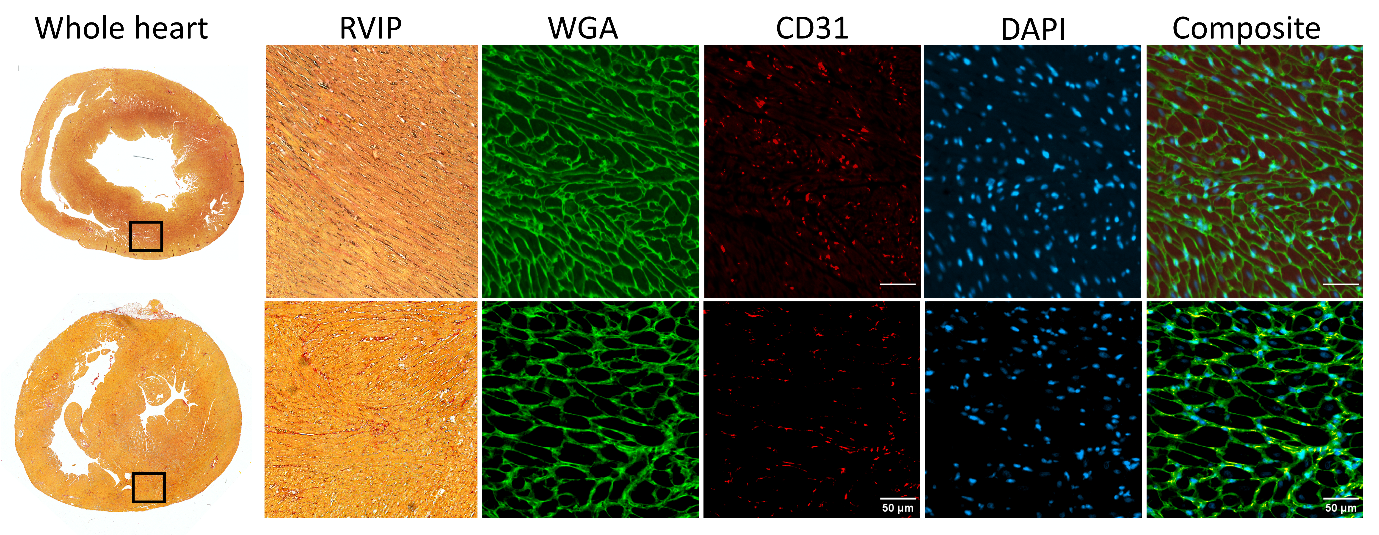


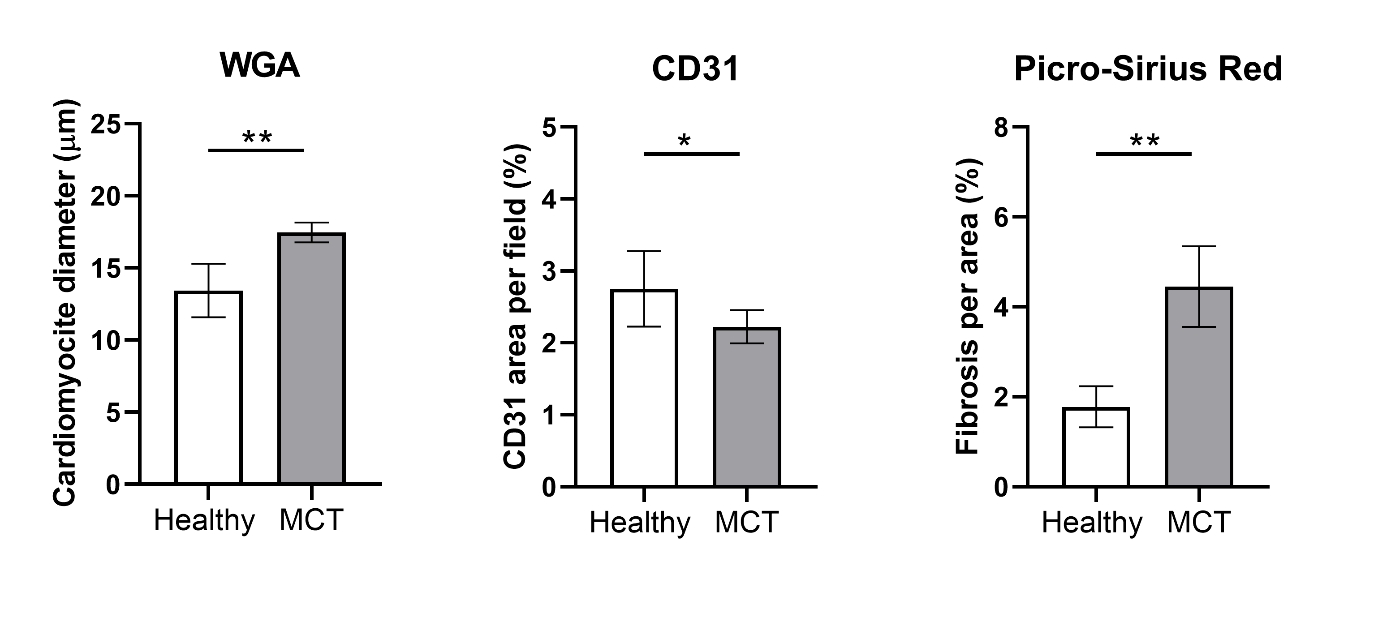


**Figure S5: Female control (n=3) and monocrotaline (MCT, n=4) histological images.** Picrosirius red stained whole heart (selection box size: 300µm × 350µm) and right ventricular insertion points (RVIP) are displayed, along with double immunofluorescence images stained for cardiomyocyte periphery (WGA), endothelial cells (CD31) and nuclei (DAPI). The immunofluorescent stained images are also shown as a composite merged image. Scale bar = 50 μm. Significance values were: * p<0.05; ** p<0.01, *** p<0.001 when compared to control group, t-test.

The MRI metrics derived from cine, flow MRI and ECV measurements, expressed as mean value ± standard deviation (SD), are presented in Table S1 for MCT males and Table S2 for SuHx males, respectively.

**Table S1:** **Cardiac MRI metrics of male rats derived from cine: healthy (n=12), 2-week MCT (n=12) and 4-week MCT (n=9), flow MRI: healthy (n=5), 2-week MCT (n=7) and 4-week MCT (n=4) and ECV scans: healthy (n=7), 2-week MCT (n=5) and 4-week MCT (n=5).** Data is presented as mean ± standard deviation (SD), one-way ANOVA - comparison to healthy, Dunnett correction for multiple variables. Significance values were: *p<0.05, **p<0.01, ***p<0.001, ****p<0.0001.

|  | **Healthy** | **2w-MCT** | **4w-MCT** |
| --- | --- | --- | --- |
| **HR (bpm)** | 348±25.2 | 312.6±14.4 | 287±24.3 |
| **BW (g)** | 210.7±18.7 | 279.5±13.8^**^ | 297.8±15.3^**^ |
| **LVEF (%)** | 59.40±5.7 | 61.32±2.6 | 57.81±6.4 |
| **RVEF (%)** | 59.39±3.9 | 54.13±3.9 | 39.73±16.5^***^ |
| **BSA (cm^2^)** | 347.79±21.2 | 420.12±13.8 | 438.31±14.9 |
| **LVEDVI (μl/cm^2^)** | 1.45±0.1 | 1.24±0.1^****^ | 0.69±0.1^****^ |
| **LVMI (mg/cm^2^)** | 1.22±0.06 | 1.19±0.08 | 1.31±0.1 |
| **RVEDVI (μl/cm^2^)** | 1.19±0.14 | 1.18±0.1 | 1.66±0.2^****^ |
| **RVMI (mg/cm^2^)** | 0.543±0.03 | 0.61±0.04 | 0.88±0.07^**^ |
| **LVESVI (μl/cm^2^)** | 0.59±0.1 | 0.48±0.08 | 0.29±0.06^****^ |
| **RVESVI (μl/cm^2^)** | 0.52±0.1 | 0.54±0.07 | 1.09±0.3^****^ |
| **LVSVI (μl/cm^2^)** | 0.85±0.02 | 0.76±0.08^*^ | 0.37±0.07^****^ |
| **RVSVI (μl/cm^2^)** | 0.67±0.05 | 0.64±0.09 | 0.48±0.1^***^ |
| **RVCI (μl/min/m^2^)** | 0.23±0.02 | 0.19±0.02 | 0.15±0.05^****^ |
| **LVCI (μl/min/m^2^)** | 0.29±0.02 | 0.23±0.02^**^ | 0.11±0.01^****^ |
| **VMI** | 0.45±0.01 | 0.50±0.05 | 0.68±0.09^****^ |
| **RVESV/RVSV(Eea/Ees*)** | 1.38±0.3 | 1.19±0.2 | 0.49±0.2^****^ |
| **RV-ECV (%)** | 16.44±3.4 | 22.76±6.5 | 26.21±8.4^**^ |
| **LV-ECV (%)** | 14.56±4.2 | 18.36±4.3 | 26.48±2.2^**^ |
| **IS-ECV (%)** | 17.66±4.6 | 20.24±1.7 | 30.95±5.3^***^ |
| **RV-IP (%)** | 15.18±3.7 | 21.10±7.2 | 35.79±6.4^****^ |
| **PAAT (%RR)** | 0.20±0.01 | 0.17±0.03^***^ | 0.104±0.03^****^ |
| **PTN (%RR)** | 0.19±0.07 | 0.22±0.05 | 0.11±0.06^****^ |
| **TTN (%RR)** | 0.34±0.08 | 0.39±0.04^**^ | 0.215±0.1^****^ |
| **ES (%RR)** | 0.57±0.03 | 0.58±0.03 | 0.63±0.07 |
| **Slope (×10^3^)** | -7.3±2.1 | -4.8±0.7^**^ | -12.1±6.1^**^ |
| **PTES (%RR)** | 0.372±0.03 | 0.411±0.03 | 0.526±0.09 |

**Table S2:** **Cardiac MRI metrics of male rats derived from cine: 4-week SuHx (n=7) and 6-week SuHx (n=15), 8-week SuHx (n=10), 10-week SuHx (n=10), flow MRI: : 4-week SuHx (n=7) and 6-week SuHx (n=7), 8-week SuHx (n=5), 10-week SuHx (n=5), and ECV scans: 6-week SuHx (n=7), 8-week SuHx (n=5), 10-week SuHx (n=5).** Data is presented as mean ± standard deviation (SD), one-way ANOVA - comparison to healthy, Dunnett correction for multiple variables. Significance values were: *p<0.05, **p<0.01, ***p<0.001, ****p<0.0001.

|  | **4w-SuHx** | **6w-SuHx** | **8w-SuHx** | **10w-Suhx** |
| --- | --- | --- | --- | --- |
| **HR (bpm)** | 313±29.6 | 298.5±26.2 | 335±13.3 | 273.7±40.4 |
| **BW (g)** | 289±18.1^**^ | 344.2±22.3^***^ | 384.6±28.9^***^ | 428±29.6^***^ |
| **LVEF (%)** | 60.78±10.8 | 60.04±4.5 | 59.11±4.3 | 56.25±4.1 |
| **RVEF (%)** | 28.63±3.8^***^ | 31.88±7.1^****^ | 31.04±7.9^****^ | 36.56±16.05^****^ |
| **BSA (cm^2^)** | 434.6±16.9 | 482.6±20.8 | 519.6±25.8 | 558.05±25.7 |
| **LVEDVI (μl/cm^2^)** | 0.72±0.1^****^ | 0.91±0.1^****^ | 0.94±0.1^****^ | 0.96±0.1^****^ |
| **LVMI (mg/cm^2^)** | 1.61±0.07^*^ | 1.22±0.1 | 1.23±0.08 | 1.09±0.1 |
| **RVEDVI (μl/cm^2^)** | 1.45±0.06^****^ | 1.67±0.08^****^ | 1.57±0.09^****^ | 1.54±0.3^***^ |
| **RVMI (mg/cm^2^)** | 1.05±0.07^****^ | 0.95±0.04^****^ | 0.90±0.06^****^ | 0.78±0.1^***^ |
| **LVESVI (μl/cm^2^)** | 0.29±0.09^****^ | 0.36±0.05^****^ | 0.38±0.04^****^ | 0.42±0.02^***^ |
| **RVESVI (μl/cm^2^)** | 1.04±0.09^****^ | 1.14±0.1^****^ | 1.09±0.1^****^ | 1.01±0.4^****^ |
| **LVSVI (μl/cm^2^)** | 0.43±0.04^****^ | 0.54±0.08^****^ | 0.56±0.08^****^ | 0.54±0.1^****^ |
| **RVSVI (μl/cm^2^)** | 0.45±0.05^*^ | 0.53±0.1^*^ | 0.48±0.1^**^ | 0.52±0.1^*^ |
| **RVCI (μl/min/m^2^)** | 0.148±0.03^***^ | 0.16±0.04^****^ | 0.162±0.03^**^ | 0.14±0.05^****^ |
| **LVCI (μl/min/m^2^)** | 0.13±0.01^****^ | 0.16±0.02^****^ | 0.19±0.02^****^ | 0.14±0.04^****^ |
| **VMI** | 0.712±0.1^****^ | 0.78±0.04^****^ | 0.74±0.05^****^ | 0.71±0.08^****^ |
| **RVESV/RVSV (Eea/Ees*)** | 0.42±0.04^****^ | 0.48±0.1^****^ | 0.47±0.1^****^ | 0.65±0.3^****^ |
| **RV-ECV (%)** |  | 17.20±2.04 | 20.97±3.6 | 23.96±0.72^*^ |
| **LV-ECV (%)** |  | 17.89±1.4 | 22.54±1.9^*^ | 19.15±1.3 |
| **IS-ECV (%)** |  | 23.13±4.08 | 29.93±7.8^**^ | 28.23±5.3^**^ |
| **RV-IP (%)** |  | 23.25±2.1^***^ | 28.01±3.7^**^ | 22.58±0.7^*^ |
| **PAAT (%RR)** | 0.049±0.02^****^ | 0.055±0.02^****^ | 0.061±0.03^****^ | 0.055±0.02^****^ |
| **PTN (%RR)** | 0.088±0.02^***^ | 0.08±0.01^***^ | 0.087±0.01^***^ | 0.066±0.02^***^ |
| **TTN (%RR)** | 0.138±0.03^****^ | 0.14±0.02^****^ | 0.148±0.03^****^ | 0.122±0.01^****^ |
| **ES (%RR)** | 0.415±0.07 | 0.407±0.08 | 0.430±0.04 | 0.402±0.06 |
| **Slope (×10^3^)** | -9.8.1±3.2 | -9.6.3±3.03 | -10.4.8±3.8 | -11.7.9±3.07^*^ |
| **PTES (%RR)** | 0.366±0.05 | 0.351±0.07 | 0.369±0.02 | 0.345±0.05 |
|  |  |  |  |  |

The MRI metrics derived from cine measurements for females MCT and SuHx rats, expressed as mean value ± standard deviation (SD), are presented in Table S3.

**Table S3: Cardiac MRI metrics of female rats derived from cine scans: healthy (n=5), 2-week MCT (n=5), 4-week MCT (n=5), 6-week SuHx (n=6) and 8-week SuHx (n=6).** Data is presented as mean ± standard deviation (SD), one-way ANOVA - comparison to healthy, Dunnett correction for multiple variables. Significance values were: *p<0.05, **p<0.01, ***p<0.001, ****p<0.0001.

|  | **Healthy** | **2-weekMCT** | **4-week MCT** | **6-week SuHx** | **8-week SuHx** |
| --- | --- | --- | --- | --- | --- |
| **BW (g)** | 206±6.3 | 226±5.3^***^ | 238±4.8^***^ | 283±10.4^***^ | 280±15^***^ |
| **HR (bpm)** | 384±17.5 | 378±29 | 388±29.2 | 238±17.5 | 344±36.3 |
| **LVEF (%)** | 70.42±1.58 | 65.08±1.6^**^ | 63.82±2.36^**^ | 62.21±2.76^*^ | 57.26±5.79^***^ |
| **RVEF (%)** | 74.57±2.8 | 69.68±3.3 | 50.4±10.1^***^ | 52.47±3.9^**^ | 48.50±13.8^***^ |
| **LVEDVI (μl/cm^2^)** | 1.12±0.07 | 1.12±0.05 | 0.86±0.16^**^ | 1.03±0.08 | 1.01±0.19 |
| **LVMI (mg/cm^2^)** | 1.01±0.03 | 0.99±0.02 | 0.97±0.01 | 1.08±0.04 | 1.16±0.12^*^ |
| **RVEDVI (μl/cm^2^)** | 0.92±0.11 | 0.84±0.05 | 0.97±0.08 | 1.15±0.09^*^ | 1.18±0.17^*^ |
| **RVMI (mg/cm^2^)** | 0.47±0.04 | 0.45±0.01 | 0.58±0.02^***^ | 0.67±0.05^*^ | 0.72±0.17^*^ |
| **LVESVI (μl/cm^2^)** | 0.33±0.03 | 0.39±0.01 | 0.31±0.04 | 0.38±0.04 | 0.42±0.06^*^ |
| **RVESVI (μl/cm^2^)** | 0.23±0.02 | 0.25±0.03 | 0.48±0.12^***^ | 0.54±0.08^*^ | 0.62±0.27^**^ |
| **LVSVI (μl/cm^2^)** | 0.79±0.04 | 0.73±0.05 | 0.55±0.12^***^ | 0.64±0.06 | 0.58±0.14^**^ |
| **RVSVI (μl/cm^2^)** | 0.69±0.10 | 0.58±0.03 | 0.49±0.09^**^ | 0.60±0.03 | 0.55±0.11 |
| **RVCI (μl/min/m^2^)** | 0.26±0.03 | 0.22±0.02 | 0.19±0.02^***^ | 0.20±0.02^*^ | 0.19±0.05^*^ |
| **LVCI (μl/min/m^2^)** | 0.79±0.01 | 0.27±0.02 | 0.21±0.04^***^ | 0.21±0.03^*^ | 0.20±0.05^**^ |
| **VMI** | 0.47±0.04 | 0.45±0.01 | 0.59±0.03^***^ | 0.62±0.04^**^ | 0.61±0.08^**^ |
| **RVESV/RVSV (Eea/Ees*)** | 2.97±0.49 | 2.33±0.37 | 1.09±0.47^***^ | 1.11±0.16^***^ | 1.04±0.46^***^ |

**Original western blot data for Fig 7A and 7B**

1. **Uncropped scan of western blot gels of MCT** **(RV tissue)**


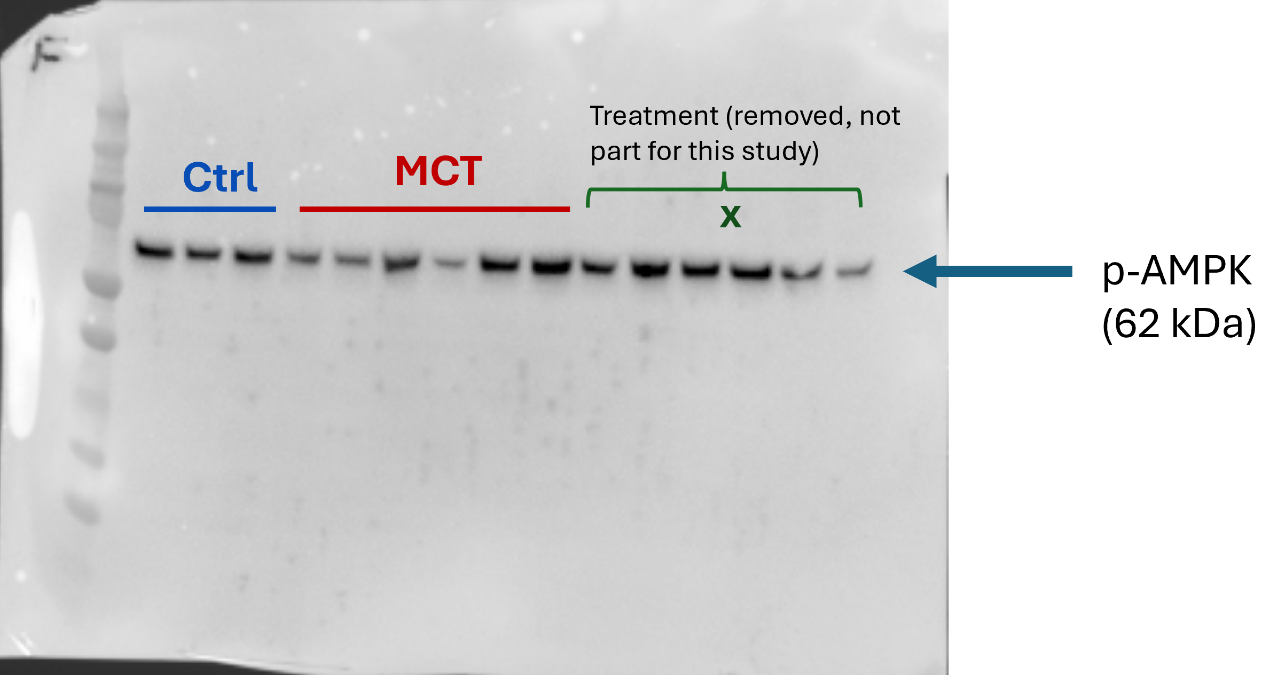


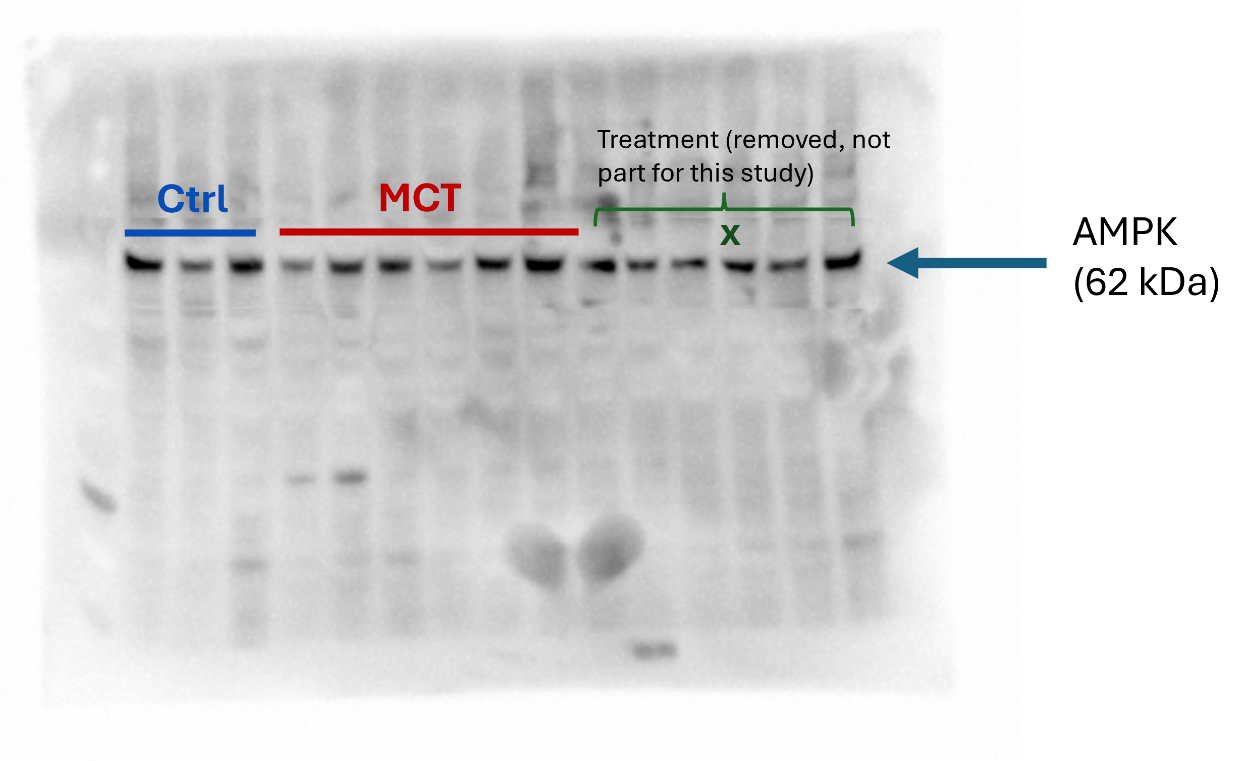


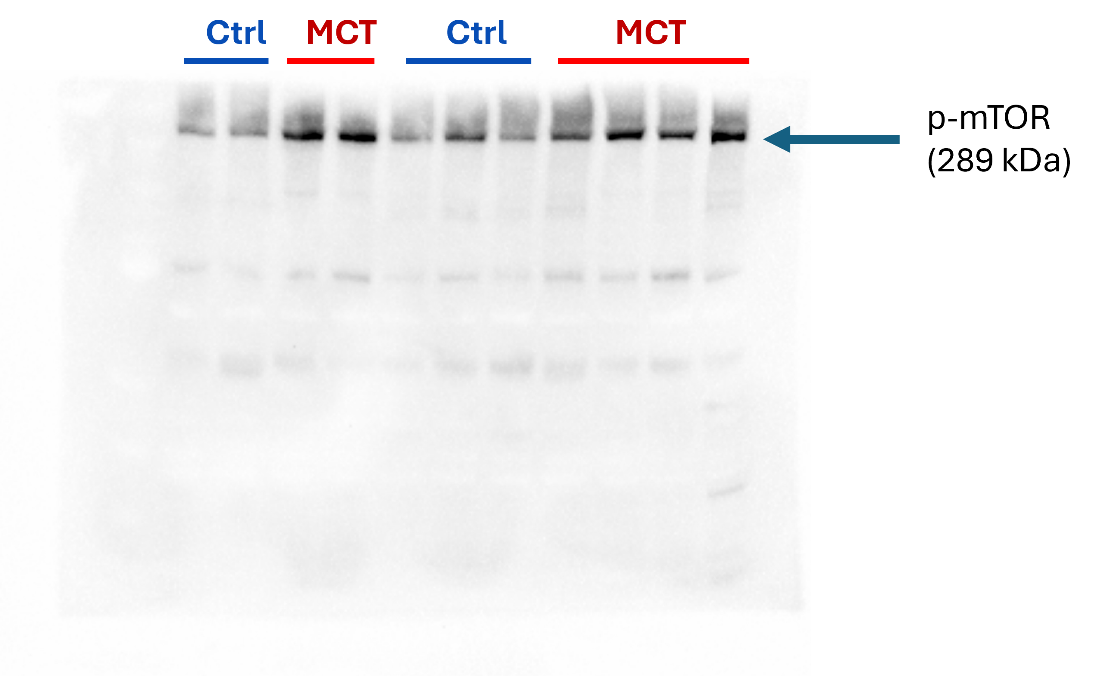


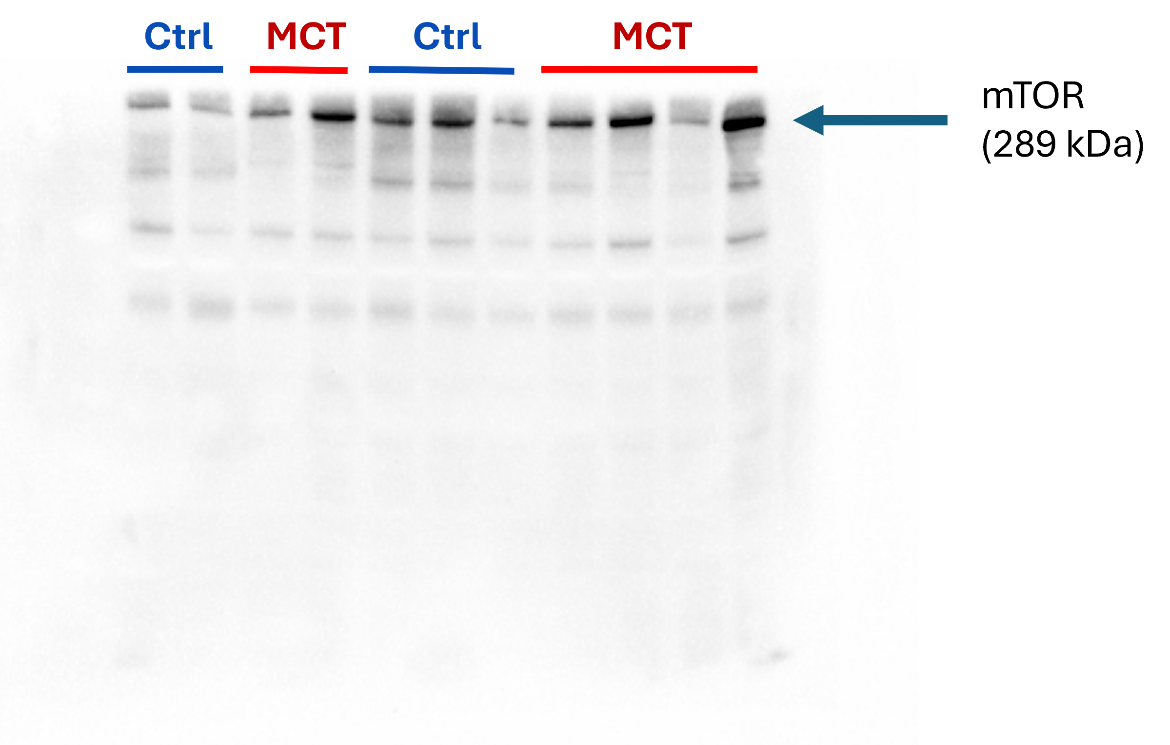


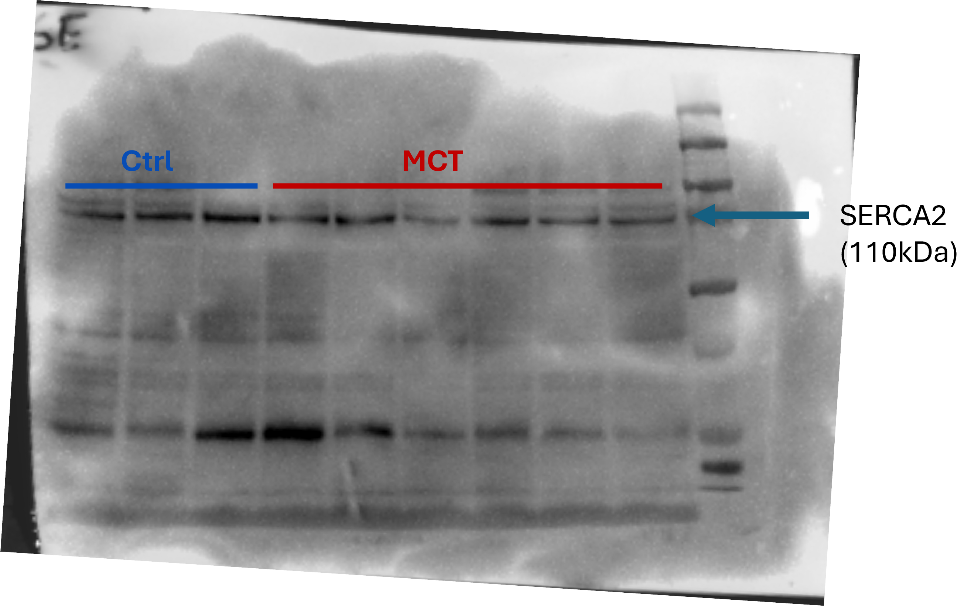


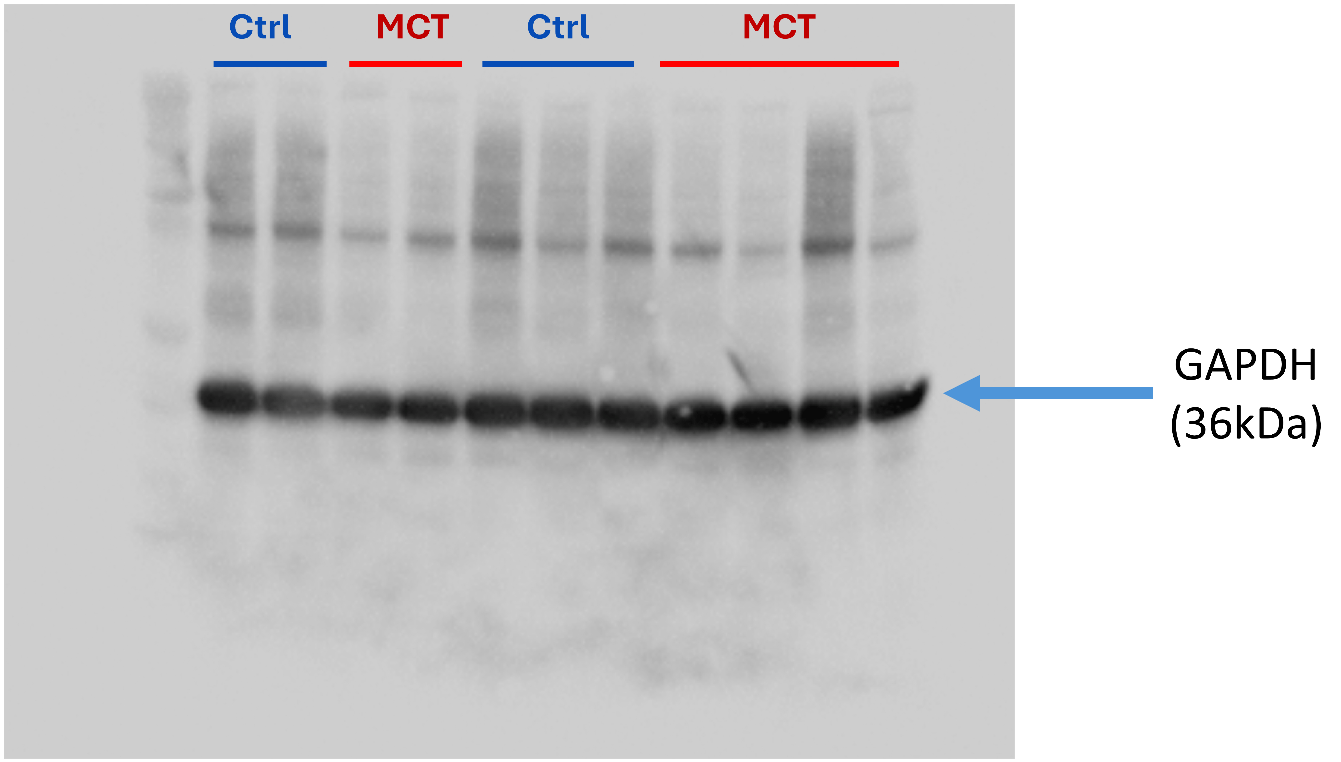


1. **Uncropped scan of western blot gels of SuHx** **(RV tissue)**


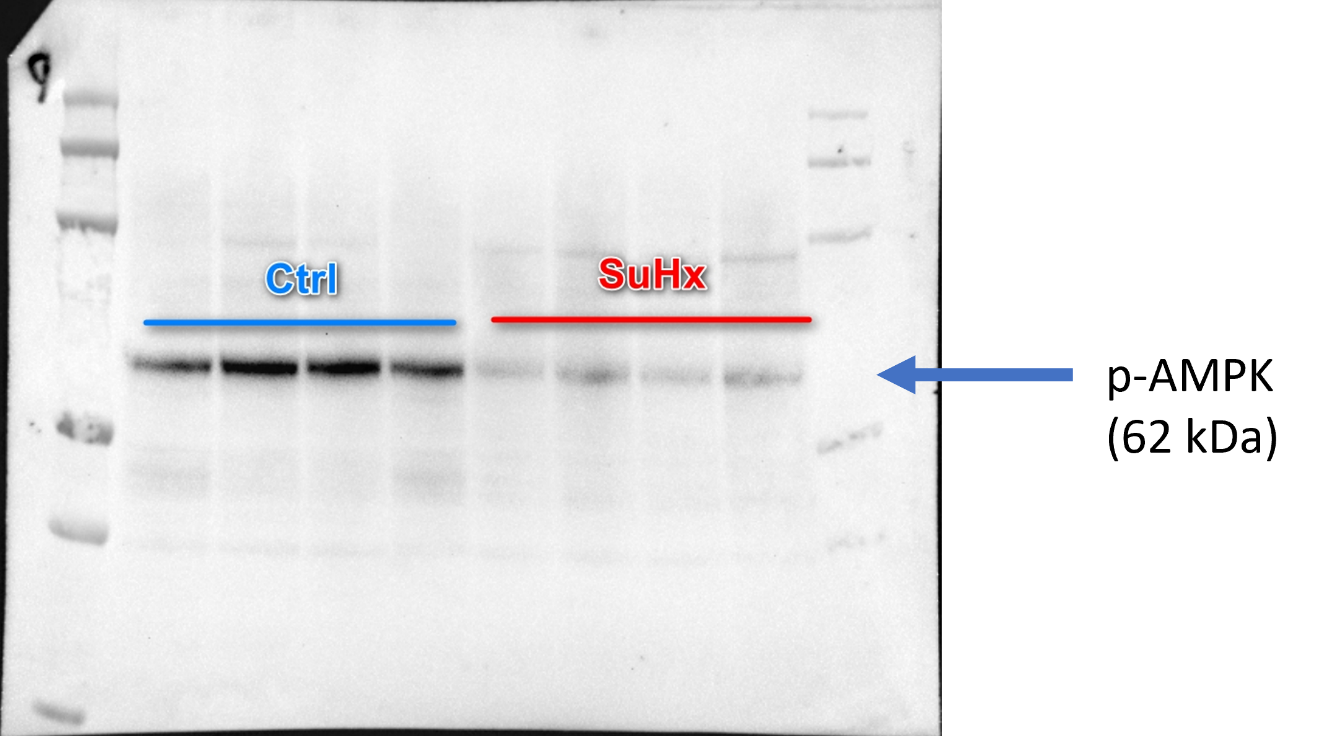


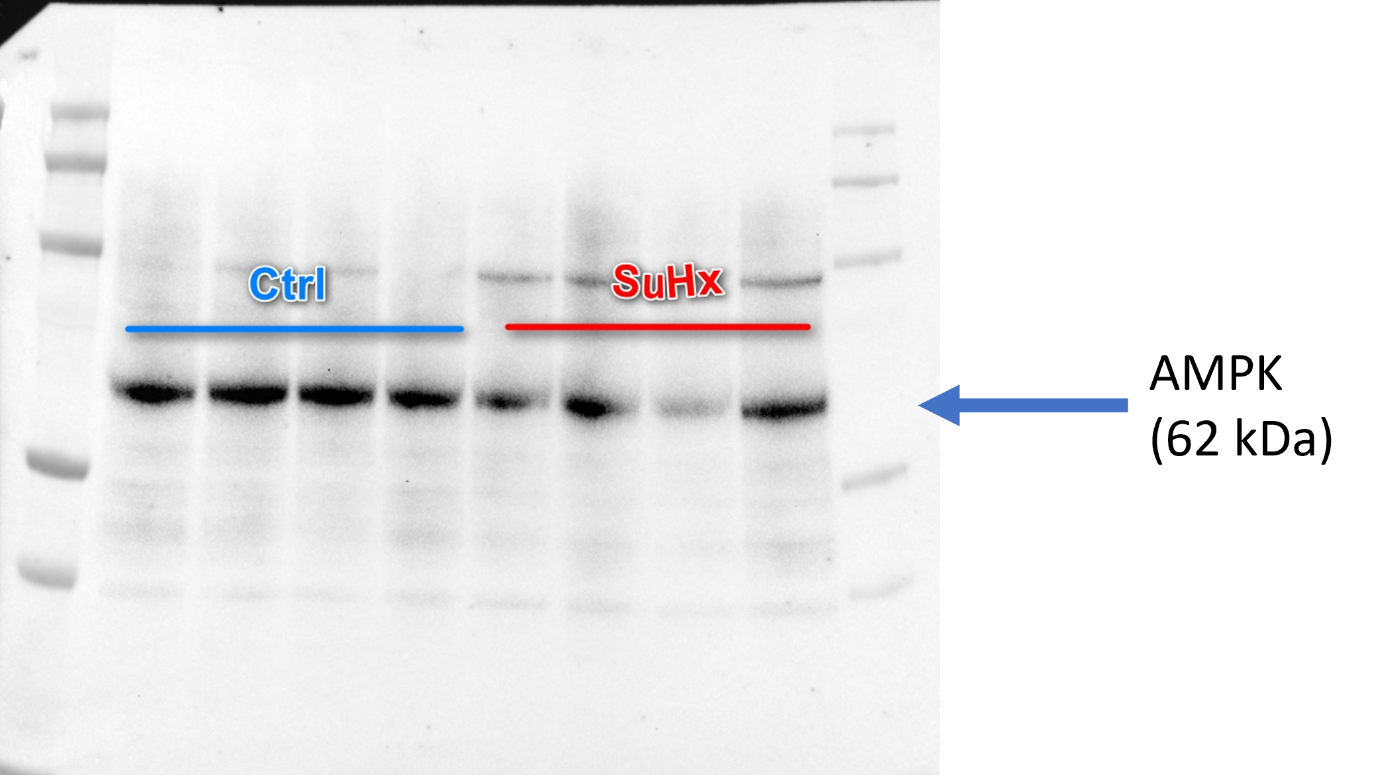


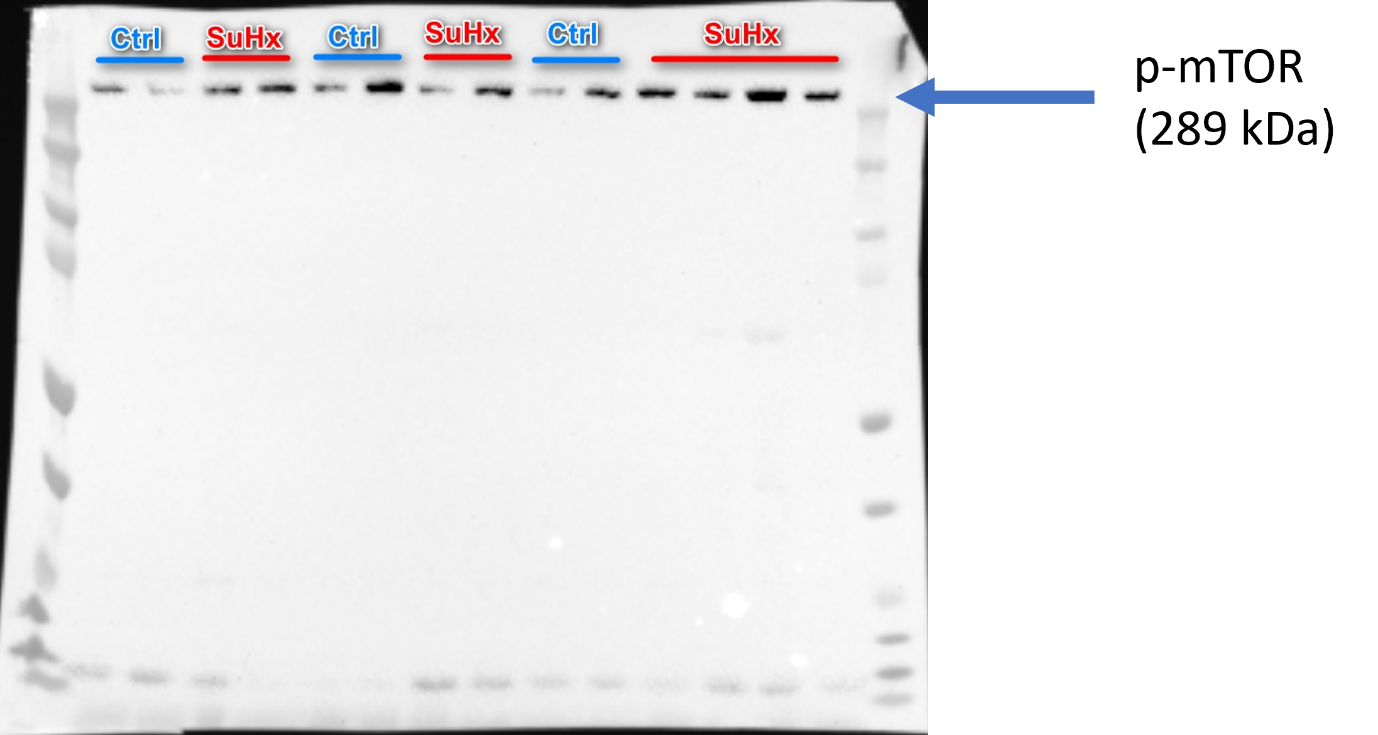


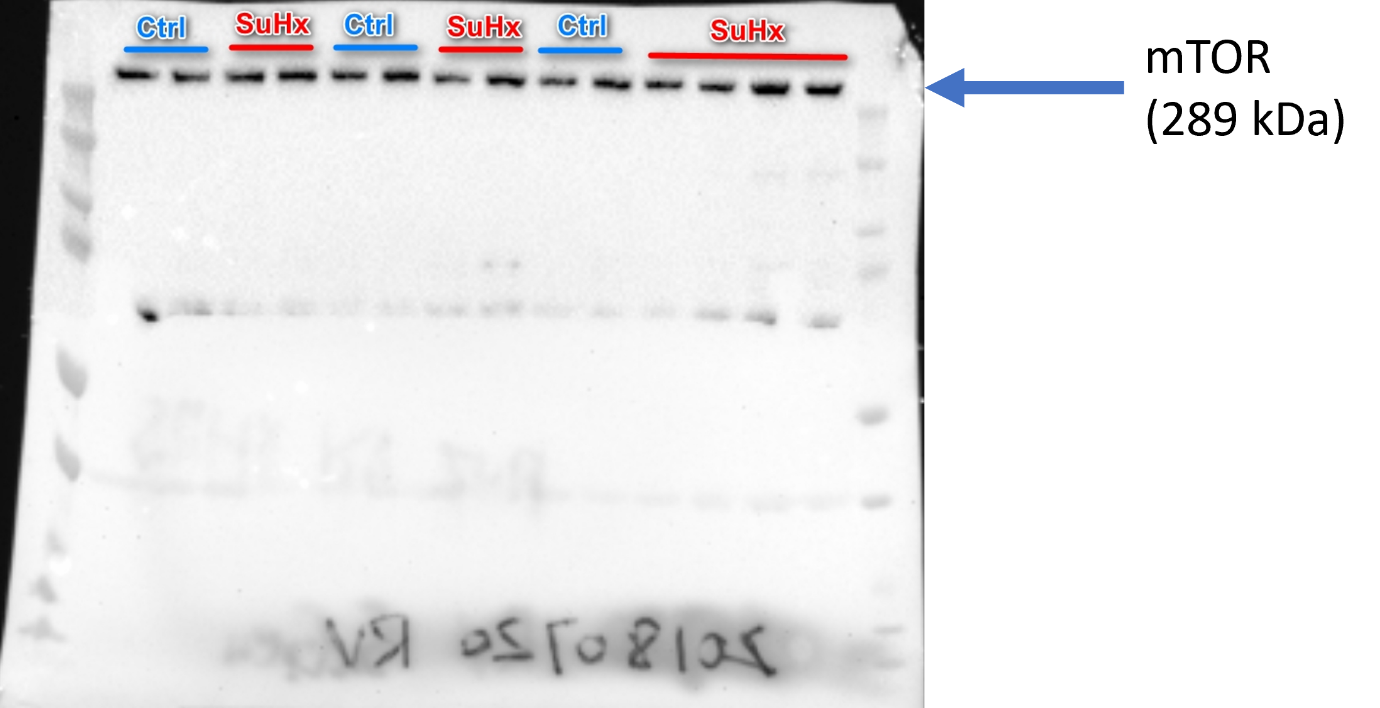


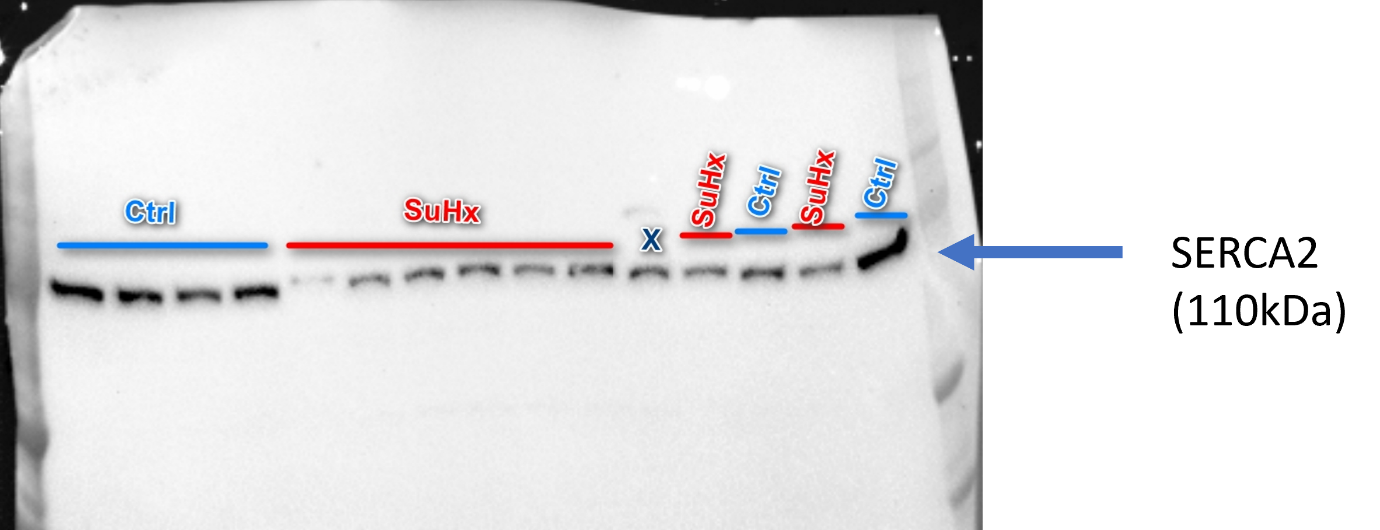


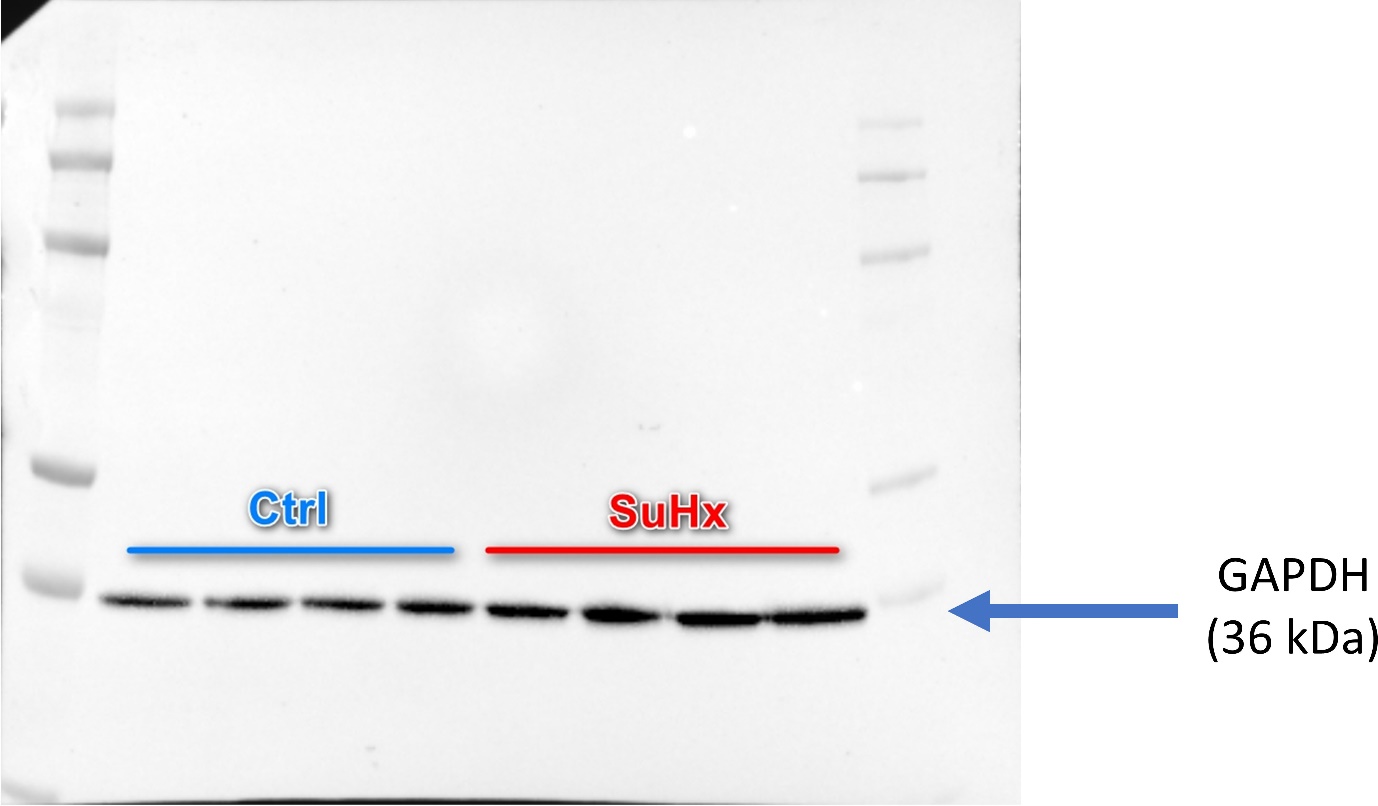

Supplement: Supplementary file 1 — Supplementary Information. [file 41598_2024_79029_MOESM1_ESM.docx]
